# A symbolic network-based nonlinear theory for dynamical systems observability

**DOI:** 10.1038/s41598-018-21967-w

**Published:** 2018-02-28

**Authors:** Christophe Letellier, Irene Sendiña-Nadal, Ezequiel Bianco-Martinez, Murilo S. Baptista

**Affiliations:** 1 CORIA-UMR 6614 Normandie Université, Campus Universitaire du Madrillet, F-76800 Saint-Etienne du Rouvray, France; 2 Complex Systems Group, Universidad Rey Juan Carlos, 28933 Móstoles, Madrid Spain; 3 Center for Biomedical Technology, Universidad Politécnica de Madrid, 28223 Pozuelo de Alarcón, Madrid Spain; 4 Institute for Complex Systems and Mathematical Biology, SUPA, University of Aberdeen, Old Aberdeen, AB24 3UE United Kingdom

## Abstract

When the state of the whole reaction network can be inferred by just measuring the dynamics of a limited set of nodes the system is said to be fully observable. However, as the number of all possible combinations of measured variables and time derivatives spanning the reconstructed state of the system exponentially increases with its dimension, the observability becomes a computationally prohibitive task. Our approach consists in computing the observability coefficients from a symbolic Jacobian matrix whose elements encode the linear, nonlinear polynomial or rational nature of the interaction among the variables. The novelty we introduce in this paper, required for treating large-dimensional systems, is to identify from the symbolic Jacobian matrix the minimal set of variables (together with their time derivatives) candidate to be measured for completing the state space reconstruction. Then symbolic observability coefficients are computed from the symbolic observability matrix. Our results are in agreement with the analytical computations, evidencing the correctness of our approach. Its application to efficiently exploring the dynamics of real world complex systems such as power grids, socioeconomic networks or biological networks is quite promising.

## Introduction

Variables spanning the state space of a dynamical system which is irreducible to a few smaller subsystems are always dependent on each other through linear and nonlinear interactions. Consequently, one may expect to be able to determine an adequate subset of variables together with their well-selected Lie derivatives to get a full observability of the underlying dynamics, that is, for distinguishing all possible states of the network^1,2^. With the emergence of the Science of Complexity, complex networks are more and more often considered in various fields as well exemplified by power grids^3^, socio-economics networks^4–6^, or biological systems^7–10^. To allow a reliable monitoring, dynamical analysis or control of these high-dimensional systems, suitable and systematic techniques are required to identify the subset of variables providing the best (if not the full) observability of their underlying dynamics. A related problem is how to unfold the whole dynamics by completing this subset of variables to reconstruct a space whose dimension is at least equal to the dimension of the original state space.

Dealing with multivariate time series, specially those produced by high-dimensional dynamical networks, is not a trivial problem^11–13^. Attempts to estimate network observability using symbolic techniques^14,15^ were made to overcome the large computational times associated with the exact analytical calculations. In those approaches, a dimension reduction is performed in real time on a symbolic observability matrix until state estimation is possible from the selected measurements. However, linear and nonlinear interactions among variables are considered on an equal footing while it is strongly required to distinguish them for a reliable assessment of the observability of a system^2,13^. In order to tackle such a challenging task, we propose a methodological approach that will be applied to reaction networks derived from dynamical systems with appropriately large dimension to corroborate our assessments with rigorous analytical calculations, and yet provide a framework making also possible the verification of observability in networked dynamical systems. The chosen reaction networks are models of interesting biological and physical systems: the circadian oscillation in the Drosophila period protein, the Rayleigh-Bénard convection, and the DNA replication. They also represent nonlinear systems with increasing nonlinear complexity, commonly observed in other natural and man-made systems. Therefore, they are an appropriate subset of nonlinear systems to serve as testbed of our approach’s performance and reliability. In addition, we will show that our approach correctly identifies whether a nonlinear dynamical system is fully or only partially observable, an information not accurately obtained by previously proposed methodological approaches^16–19^.

Since we are dealing with dynamical systems in general, the Jacobian matrix will be used to access the nature of how variables interact, allowing us to optimize our assessment of the symbolic observability coefficients. For high-dimensional complex systems this is a quite demanding computation since the number of cases to investigate increases with the system’s dimension and exact analytical computations are prohibitive. Indeed, in practice, monitoring all the variables defining the system’s state is experimentally infeasible or inefficient, and it is of utmost importance to develop a methodological framework addressing the problem of targeting those variables yielding full observability. Despite several approaches have been proposed^16,17^, most of them neglect the nonlinear nature typically exhibited by complex systems and/or do not provide the space reconstructed from the measured variables. On the one hand, since nonlinearities are most often related to a lack of observability, linear approaches cannot properly address this problem. On the other hand, finding the appropriate combination of sensors (and time derivatives) spanning the reconstructed space is a very time demanding computational task for large dimensional systems.

Here, we adopted a nonlinear symbolic approach taking into account the nature of the interactions among variables and analyze the distribution of the linear and nonlinear load of the variables in the symbolic Jacobian matrix of the system. By means of two easy-to-implement criteria we are able to successfully identify the minimal set of variables (and their time derivatives) candidate to be measured for completing the reconstructed space. Our results are in full agreement with the analytical prediction of getting a no null determinant of the observability matrix and the technique drastically reduces the search for candidate variables, thus providing a key step to observe and model natural and man-made complex systems of large dimension.

The subsequent part of the paper is organized as follows. Section Results is devoted to illustrate how our proposed approach works considering a few large dimensional dynamical systems. Section Methods briefly introduces the observability theory, and the current challenges for the determination of a system’s observability in nonlinear systems. Finally, the Discussion section provides some conclusions to this work.

## Results

On rare occasions, nonlinear systems are fully observable from just a single scalar time series^20^ as previously investigated for many chaotic systems^21–23^. Since full (global) observability warrants that every distinct point of the original state space $\,x\in {{\mathbb{R}}}^{d}$ can be univocally identified, there is a great interest to target the minimal set of variables to measure for accomplishing such a full observability condition.

As shown in Section Methods, to assess the (local or global) observability through a given measurement vector *s*, both a subset of *m* variables–sometimes designated as “sensors”^24^ –and the Lie derivatives have to be provided. Our aim is therefore to provide a method that can indeed solve the problem of determining the minimum set of variables to measure for observing a large complex system. In order to avoid testing the rank of the observability matrix via algebraic computation, we propose to use a technique based on a symbolic computation of the observability matrix in which the terms are not explicitely expressed but only their linear, nonlinear polynomial or rational character^2,13^.

The general and systematic procedure developed by Bianco-Martinez and coworkers^2^ and compiled in the Methods section, is in fact very time consuming since the computation of the observability coefficients corresponding to all the 5.2 ⋅ 10^6^ possible vectors spanning a reconstructed space of a 13-dimensional system would require intense and long computational time (more than 18 days). One optimization strategy is to reduce the number of possible combinations by identifying candidate variables that should be disregarded as members of the measurement set. A lack of observability has its origin in the existence of a singular observability manifold, a domain in the original state space where the determinant Det ${\mathscr{O}}$ of the observability matrix ${\mathscr{O}}$ is zero^25^. Let us note here that there is one very special case in which Det ${\mathscr{O}}$ ≈ 0 and, consequently, practical problems in the state estimation may occur. This usually happens when Det ${\mathscr{O}}$ depends on some parameter(s) which may be arbitrarily small^26^. In general, a linear system may be (rarely and practically) non-observable with a nonzero determinant of the observability matrix. By construction, a null or non-constant determinant Det ${\mathscr{O}}$ is rooted in a null or a nonlinear component in the Jacobian matrix. Our technique relies precisely on tracking those nonlinear terms in the Jacobian matrix and, therefore, taking into account both linear and nonlinear interactions between variables becomes so relevant in assessing observability.

By analogy with what is done for chemical reactions^27^, it is possible to consider any dynamical system as a reaction network, whose associated weighted adjacency matrix is the symbolic Jacobian matrix $\tilde{{\mathscr{J}}}$. Using the terminology from graph theory, we define the linear out-strength of the node *i*, ${\sigma }_{{\rm{out}}}^{{\rm{lin}}}(i)$, as the number of times the *i*th variable appears in linear terms in the governing equations, that is,

1 $${\sigma }_{{\rm{out}}}^{{\rm{lin}}}(i)=\mathop{\sum _{j\ne i}}\limits_{j|{\tilde{J}}_{ji}=1}{\tilde{J}}_{ji}\mathrm{.}$$

The larger is ${\sigma }_{{\rm{out}}}^{{\rm{lin}}}(i)$, the higher the probability the *i*th variable needs not to be measured because it is related to other variables *via* linear couplings which will not induce nonlinear terms in the determinant of the observability matrix.

The situation in which ${\tilde{J}}_{ij}={\tilde{J}}_{ji}=1$ and ${\sigma }_{{\rm{out}}}^{{\rm{lin}}}(i)={\sigma }_{{\rm{out}}}^{{\rm{lin}}}(j)=1$ means that variables *i* and *j* are exclusively linearly coupled with no other variables involved. Consequently, full observability of the *i*th variable can only be accessed by measuring the *j*th variable and *vice versa*. It is thus necessary (and sufficient) to measure at least one of them because they cannot be simultaneously excluded from the set of measured variables. A second criterion to decide which variable to choose between these two is needed. The idea is built on how a variable candidate to be non-measured is influenced by the other candidate variables. Let be {*x*_*k*_} the set of variables candidate to be non measured with *k* ∈ *V*_nm_ ⊂ {1, 2, ..., *m*} and *V*_nm_ the set of integers indexing the non-measured variables. Thus, the in-strength of the *i*th variable provided by the non-measured ones is defined as

2 $${\sigma }_{{\rm{in}}}^{{\rm{nm}}}(i)=\mathop{\sum _{k\in {V}_{{\rm{nm}}}}}\limits_{k\ne i}{\tilde{J}}_{ik}$$

where ${\tilde{J}}_{ik}=1\equiv 1$, ${\tilde{J}}_{ik}=\overline{1}\equiv 2$, and ${\tilde{J}}_{ik}=\overline{\overline{1}}\equiv 3$. Using this correspondence for the symbolic terms, we can assume that the larger is ${\sigma }_{{\rm{in}}}^{{\rm{nm}}}(i)$, the less observable through the *i*th variable the system is. The rationale is as follows: the more nonlinearly coupled is the *i*th function *f*_*i*_(***x***) with the non-measured variables, the larger the degree of the determinant of the observability matrix, and the less observable the dynamics through the measurements is^22^. Therefore, we should preferably remove that variable with the largest non-measured in-strength ${\sigma }_{{\rm{in}}}^{{\rm{nm}}}$.

Therefore, the minimal set of variables to measure for reconstructing a state space with a full observability can be automatically determined from (i) the symbolic Jacobian matrix $\tilde{{\mathscr{J}}}$ of the system under study, (ii) the linear out-strength ${\sigma }_{{\rm{out}}}^{{\rm{lin}}}$, and (iii) the in-strength ${\sigma }_{{\rm{in}}}^{{\rm{nm}}}$ provided by the non-measured variables. Note that the knowledge of the exact functional dependence of the coupling between variables is not necessary, only its polynomial or fractional nature^2^. From the vector of state variables ***x***, those components *x*_*i*_ having the largest ${\sigma }_{{\rm{out}}}^{{\rm{lin}}}$ are discarded as candidates to be sensors after having checked they are present at least once in the equations governing the dynamics of a sensor variable. All remaining possible embeddings ***s*** that can be constructed from the final set of variables candidate to become sensors are then tested using a Matlab® algorithm and ranked according to the corresponding estimated symbolic observability coefficient ***η***_***s***_.

In order to validate whether our proposed method is in agreement with algebraic computations, we will consider three dynamical systems of increasing dimension (*d* = 5, 9 and 13) describing complex systems coming from biology or physics. As it is known that the presence of symmetries in the state space can affect the assesment of observability^22^, we will also consider the case of equivariant dynamical systems obeying *f*(Γ ⋅ *x*) = Γ ⋅ *f*(*x*), where Γ defines a discrete symmetry like a rotation or an inversion^28^.

### A 5D rational model for the circadian PER oscillations in Drosophila

In our attempt to consider biological or physically motivated systems, let us start with the model

3 $$\{\begin{array}{c}\displaystyle {\dot{x}}_{1}=\frac{{v}_{s}{K}_{I}^{4}}{{K}_{I}^{4}+{x}_{5}^{4}}-\frac{{v}_{m}{x}_{1}}{{K}_{m}+{x}_{1}}\\ \displaystyle {\dot{x}}_{2}={k}_{s}{x}_{1}-\frac{{V}_{1}{x}_{2}}{{K}_{1}+{x}_{2}}+\frac{{V}_{2}{x}_{3}}{{K}_{2}+{x}_{3}}\\ \displaystyle {\dot{x}}_{3}=\frac{{V}_{1}{x}_{2}}{{K}_{1}+{x}_{2}}+\frac{{V}_{4}{x}_{4}}{{K}_{4}+{x}_{4}}-{x}_{3}(\displaystyle \frac{{V}_{2}}{{K}_{2}+{x}_{3}}+\frac{{V}_{3}}{{K}_{3}+{x}_{3}})\\ \displaystyle {\dot{x}}_{4}=\frac{{V}_{3}{x}_{3}}{{K}_{3}+{x}_{3}}-{x}_{4}(\displaystyle \frac{{V}_{4}}{{K}_{4}+{x}_{4}}+{k}_{1}+\frac{{v}_{d}}{{K}_{d}+{x}_{4}})+{k}_{2}{x}_{5}\\ \displaystyle {\dot{x}}_{5}={k}_{1}{x}_{4}-{k}_{2}{x}_{5}\end{array}$$

proposed by Goldbeter for the circadian oscillation in the *Drosophila* period protein^29^. This is a five-dimensional rational model which produces a limit cycle for the parameter values initially reported^29^. This system is interesting in the sense that its complexity already presents a big challenge from the analytical point of view. The corresponding symbolic Jacobian matrix reads as

4 $${\mathop{{\mathscr{J}}}\limits^{ \sim }}_{5{\rm{D}}}=[\begin{array}{ccccc}\bar{\bar{1}} & 0 & 0 & 0 & \bar{\bar{1}}\\ 1 & \bar{\bar{1}} & \bar{\bar{1}} & 0 & 0\\ 0 & \bar{\bar{1}} & \bar{\bar{1}} & \bar{\bar{1}} & 0\\ 0 & 0 & \bar{\bar{1}} & \bar{\bar{1}} & 1\\ 0 & 0 & 0 & 1 & 1\end{array}]\,.$$

The symbolic observability coefficients corresponding to a univariate measurement *s* = *x*_*i*_ are ${\eta }_{{x}_{1}^{5}}=0.17$, ${\eta }_{{x}_{2}^{5}}=0.08$, ${\eta }_{{x}_{3}^{5}}=0.02$, ${\eta }_{{x}_{4}^{5}}=0.09$ and, ${\eta }_{{x}_{5}^{5}}=0.30$, where the notation ${x}_{i}^{5}$ refers to the vector $({x}_{i},{\dot{x}}_{i},{\ddot{x}}_{i},{\dddot{x}}_{i},{\mathop{x}\limits^{\mathrm{....}}}_{i})$ whose observability to span the state space of the original system is estimated. According to the observability coefficient values, the ranking of the variables providing better observability is

$${x}_{5}\,\vartriangleright\,{x}_{1}\,\vartriangleright\,{x}_{4}\,\vartriangleright\,{x}_{2}\,\vartriangleright\,{x}_{3}\mathrm{.}$$

This is in a rather good agreement with the analytical determinants

5 $$\{\begin{array}{c}\displaystyle {{\rm{\Delta }}}_{{x}_{1}^{5}}=\frac{256{v}_{s}^{4}{K}_{I}^{16}{k}_{1}^{3}{V}_{3}^{2}{K}_{3}^{2}{V}_{1}{K}_{1}}{{({K}_{I}^{4}+{x}_{5}^{4})}^{8}{({K}_{3}+{x}_{3})}^{4}{({K}_{1}+{x}_{2})}^{2}}\,{x}_{5}^{12};\\ {{\rm{\Delta }}}_{{x}_{2}^{5}},{{\rm{\Delta }}}_{{x}_{3}^{5}}\,{\rm{a}}{\rm{n}}{\rm{d}}\,{{\rm{\Delta }}}_{{x}_{4}^{5}}\,{\rm{w}}{\rm{h}}{\rm{e}}{\rm{r}}{\rm{e}}\,{\rm{c}}{\rm{o}}{\rm{m}}{\rm{p}}{\rm{l}}{\rm{e}}{\rm{x}}{\rm{i}}{\rm{t}}{\rm{y}}\,{\rm{e}}{\rm{x}}{\rm{c}}{\rm{e}}{\rm{e}}{\rm{d}}{\rm{s}}\,{\rm{o}}{\rm{u}}{\rm{r}}\,{\rm{c}}{\rm{o}}{\rm{m}}{\rm{p}}{\rm{u}}{\rm{t}}{\rm{a}}{\rm{t}}{\rm{i}}{\rm{o}}{\rm{n}}{\rm{a}}{\rm{l}}\,{\rm{a}}{\rm{b}}{\rm{i}}{\rm{l}}{\rm{i}}{\rm{t}}{\rm{i}}{\rm{e}}{\rm{s}};\\ \displaystyle {{\rm{\Delta }}}_{{x}_{5}^{5}}=-\frac{{k}_{1}^{4}{V}_{3}^{3}{K}_{3}^{3}{V}_{1}^{2}{K}_{1}^{2}{k}_{s}}{{({K}_{3}+{x}_{3})}^{6}{({K}_{1}+{x}_{2})}^{4}}\,,\end{array}$$

since the simpler determinant (with singularity of the 10th degree) is obtained for variable *x*_5_ providing the best observability, then variable *x*_1_ is associated with a determinant with a singularity of the 14th degree, and the three variables *x*_2_, *x*_3_ and *x*_4_ providing the poorest observability are associated with determinants too complicated to be computed with MAPLE®. The number (125) of all possible combinations of dimension 5 is still sufficiently small for allowing a systematic computation of the corresponding symbolic observability coefficients *η*_***s***_. Prior to carry out those computations, we conducted our *a priori* analysis to target the candidate variables to be discarded. The linear out-strengths are ${\sigma }_{{\rm{out}}}^{{\rm{lin}}}\mathrm{(1)}={\sigma }_{{\rm{out}}}^{{\rm{lin}}}\mathrm{(4)}={\sigma }_{{\rm{out}}}^{{\rm{lin}}}\mathrm{(5)}=1$, the two others being null. Among the off-diagonal terms of the symbolic Jacobian matrix which are equal to 1, we have *J*_45_ = *J*_54_ = 1, meaning that at least one of the two variables *x*_4_ and *x*_5_ has to be measured. Therefore, this suggests that the sets with the minimum number of sensors providing full observability comprise at least three variables, either (*x*_2_, *x*_3_, *x*_4_) or (*x*_2_, *x*_3_, *x*_5_). In order to have a five dimensional space, these two sets have to be completed with two Lie derivatives. It turns out that only two combinations yielded full observability: $({\eta }_{{x}_{2}^{2}{x}_{3}{x}_{4}^{2}}=1)$ and $({\eta }_{{x}_{2}^{2}{x}_{3}{x}_{5}^{2}}=1)$ where the derivatives of *x*_2_ and *x*_4_ and of *x*_2_ and *x*_5_ are, respectively, the ones completing each set of sensors. These results are algebraically confirmed by the constant determinants of the observability matrix (or equivalently of the Jacobian matrix of the coordinate transformation between the original state sapce and the reconstructed space^30^) and equal to ${{\rm{\Delta }}}_{{x}_{2}^{2}{x}_{3}{x}_{4}^{2}}=-{k}_{s}{k}_{2}$ and ${{\rm{\Delta }}}_{{x}_{2}^{2}{x}_{3}{x}_{5}^{2}}={k}_{s}{k}_{1}$, respectively. The relevance of correctly chosing the derivatives is exemplified by replacing in $\,s=({x}_{2},{\dot{x}}_{2},{x}_{3},{x}_{5},{\dot{x}}_{5})$ the derivative of the fifth variable by the derivative of the third one: the coordinate transformation ${{\rm{\Phi }}}_{{x}_{2}^{2}{x}_{3}^{2}{x}_{5}}$ yields a symbolic observability coefficient ${\eta }_{{x}_{2}^{2}{x}_{3}^{2}{x}_{5}}=0.70$, thus reflecting a significant lack of observability. This is further supported by the corresponding determinant

6 $${{\rm{\Delta }}}_{{x}_{2}^{2}{x}_{3}^{2}{x}_{5}}=-\frac{{k}_{s}{V}_{4}{K}_{4}}{{({K}_{4}+{x}_{4})}^{2}}\,$$

which is now no longer constant as it depends on variable *x*_4_. There is thus a singular observabiliy manifold. On the other hand, if the derivative of the second variable is substituted with the derivative of the third one, the Jacobian matrix *J* of such a transformation ${{\rm{\Phi }}}_{{x}_{2}{x}_{3}^{2}{x}_{5}^{2}}$ can be rank deficient with a null determinant for some domain of the original state space.

Finally, we wanted to assess the observability of the couple of variables {*x*_1_,*x*_5_}, identified as sensors of the system (3) in a previous work^16^. Surprisingly enough, we got ${\eta }_{{x}_{1}{x}_{5}^{4}}=0.48$ and ${\eta }_{{x}_{1}^{2}{x}_{5}^{3}}={\eta }_{{x}_{1}^{3}{x}_{5}^{2}}={\eta }_{{x}_{1}^{4}{x}_{5}}=0$ for all the possible 5 dimensional vectors constructed with those two variables. These symbolic observability coefficients are also in agreement with the determinants of the Jacobian matrices of the corresponding transformations,

7 $$\begin{array}{rr}{{\rm{\Delta }}}_{{x}_{1}{x}_{5}^{4}}\,= & {k}_{1}^{3}\frac{{V}_{3}^{2}{K}_{3}^{2}{V}_{1}{K}_{1}}{{({K}_{3}+{x}_{3})}^{4}{({K}_{1}+{x}_{2})}^{2}}\end{array}$$

whose rational dependence on variables *x*_2_ and *x*_3_ defines a singular observability manifold associated with the transformation ${{\rm{\Phi }}}_{{x}_{1}{x}_{5}^{4}}$, and the other three determinants ${{\rm{\Delta }}}_{{x}_{1}^{2}{x}_{5}^{3}}={{\rm{\Delta }}}_{{x}_{1}^{3}{x}_{5}^{2}}={{\rm{\Delta }}}_{{x}_{1}^{4}{x}_{5}}=0$, characterizing a rank deficient observability matrix ${\mathscr{O}}$. This is therefore a first evidence that our method to reduce the number of sensor variables correctly assesses the observability of this rather complex reaction network.

### A 9D system for the Rayleigh-Bénard convection

Let us consider now a nine-dimensional system describing the dynamics of three-dimensional fluid cells with a square plateform in a Rayleigh-Bénard convection^31^. It was obtained by applying a triple second-order Fourier series ansatz to the governing hydrodynamic equations. The equations read as

8 $$\{\begin{array}{cc}{\dot{x}}_{1}= & -\sigma {b}_{1}{x}_{1}-{x}_{2}{x}_{4}+{b}_{4}{x}_{4}^{2}+{b}_{3}{x}_{3}{x}_{5}-\sigma {b}_{2}{x}_{7}\\ \displaystyle {\dot{x}}_{2}= & -\sigma {x}_{2}+{x}_{1}{x}_{4}-{x}_{2}{x}_{5}+{x}_{4}{x}_{5}-\frac{\sigma {x}_{9}}{2}\\ {\dot{x}}_{3}= & -\sigma {b}_{1}{x}_{3}+{x}_{2}{x}_{4}-{b}_{4}{x}_{2}^{2}-{b}_{3}{x}_{1}{x}_{5}+\sigma {b}_{2}{x}_{8}\\ \displaystyle {\dot{x}}_{4}= & -\sigma {x}_{4}-{x}_{2}{x}_{3}-{x}_{2}{x}_{5}+{x}_{4}{x}_{5}+\frac{\sigma {x}_{9}}{2}\\ \displaystyle {\dot{x}}_{5}= & -\sigma {b}_{5}{x}_{5}+\frac{{x}_{2}^{2}}{2}-\frac{{x}_{4}^{2}}{2}\\ {\dot{x}}_{6}= & -{b}_{6}{x}_{6}+{x}_{2}{x}_{9}-{x}_{4}{x}_{9}\\ {\dot{x}}_{7}= & -{b}_{1}{x}_{7}-R{x}_{1}+2{x}_{5}{x}_{8}-{x}_{4}{x}_{9}\\ {\dot{x}}_{8}= & -{b}_{1}{x}_{8}+R{x}_{3}-2{x}_{5}{x}_{7}+{x}_{2}{x}_{9}\\ {\dot{x}}_{9}= & -{x}_{9}-R{x}_{2}+R{x}_{4}-2{x}_{2}{x}_{6}+2{x}_{4}{x}_{6}+{x}_{4}{x}_{7}-{x}_{2}{x}_{8}\end{array}$$

where *R* is the reduced Rayleigh number and parameters *b*_*i*_ (*i* = 1, …, 6) define the geometry of the square cell^31^. This system is equivariant under the rotation (see page 506 in Gilmore and Letellier’s book^32^)

9 $${\rm{\Gamma }}=[\begin{array}{ccccccccc}0 & 0 & 1 & 0 & 0 & 0 & 0 & 0 & 0\\ 0 & 0 & 0 & 1 & 0 & 0 & 0 & 0 & 0\\ 1 & 0 & 0 & 0 & 0 & 0 & 0 & 0 & 0\\ 0 & 1 & 0 & 0 & 0 & 0 & 0 & 0 & 0\\ 0 & 0 & 0 & 0 & 0 & 0 & 0 & 0 & 1\\ 0 & 0 & 0 & 0 & 0 & 1 & 0 & 0 & 0\\ 0 & 0 & 0 & 0 & 0 & 0 & 0 & 1 & 0\\ 0 & 0 & 0 & 0 & 0 & 0 & 1 & 0 & 0\\ 0 & 0 & 0 & 0 & 1 & 0 & 0 & 0 & 0\end{array}]\,.$$

**Figure 1:**
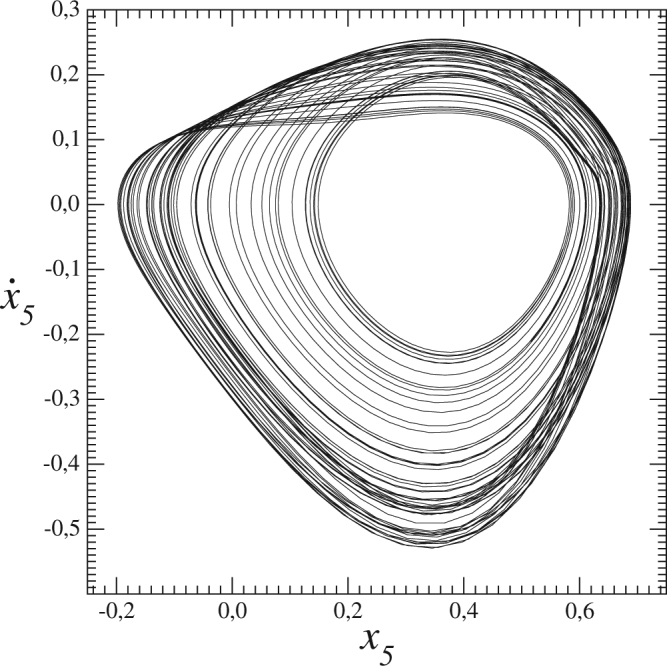
A chaotic attractor produced by the 9-dimensional dynamical network (8). Parameter values: *σ* = 0.5 and *R* = 14.22, and rest of parameter values are listed in (10).

In fact, eight variables are symmetry-related by pairs, namely (*x*_1_ − *x*_3_), (*x*_2_ − *x*_4_), (*x*_5_ − *x*_9_), and (*x*_7_ − *x*_8_), and variable *x*_6_ is the single one left invariant under the symmetry (9). Up to four co-existing chaotic attractors were observed in this system^31^. An example of one of those chaotic attractors is shown in Fig. 1 for the *b*_*i*_-values

10 $$\{\begin{array}{ccc}{b}_{1}=4\frac{1+{a}^{2}}{1+2{a}^{2}} & {b}_{2}=\frac{1+2{a}^{2}}{2(1+{a}^{2})} & {b}_{3}=2\frac{1-{a}^{2}}{1+{a}^{2}}\\ {b}_{4}=\frac{{a}^{2}}{1+{a}^{2}} & {b}_{5}=\frac{8{a}^{2}}{1+2{a}^{2}} & {b}_{6}=\frac{4}{1+2{a}^{2}}.\end{array}$$

And *a* = 0.5. This system is irreducible in the sense that it cannot be split in lower-dimensional independent systems.

This system is an interesting example because it constitutes a highly connected reaction network for which a graphical approach as the one developed by Liu and coworkers^17^ leads to only measure one of its nine variables to estimate its states (see the Supplementary Section S1).The variable that least influences the others (or equivalently, the one least “seen” by the rest) is *x*_6_ (*σ*_out_(6) = 2) since it only affects nonlinearly the derivative of *x*_9_. From a symmetry point of view, variable *x*_6_ must be measured to recover the right symmetry property: without this variable, the reconstructed state space would be necessarily associated with an inversion symmetry (a symmetry the original system does not have).

The symbolic Jacobian matrix of system (8) is

11 $${\mathop{{\mathscr{J}}}\limits^{ \sim }}_{9{\rm{D}}}=[\begin{array}{ccccccccc}1 & \bar{1} & \bar{1} & \bar{1} & \bar{1} & 0 & 1 & 0 & 0\\ \bar{1} & \bar{1} & 0 & \bar{1} & \bar{1} & 0 & 0 & 0 & 1\\ \bar{1} & \bar{1} & 1 & \bar{1} & \bar{1} & 0 & 0 & 1 & 0\\ 0 & \bar{1} & \bar{1} & \bar{1} & \bar{1} & 0 & 0 & 0 & 1\\ 0 & \bar{1} & 0 & \bar{1} & 1 & 0 & 0 & 0 & 0\\ 0 & \bar{1} & 0 & \bar{1} & 0 & 0 & 0 & 0 & \bar{1}\\ 1 & 0 & 0 & \bar{1} & \bar{1} & 1 & 1 & \bar{1} & \bar{1}\\ 0 & \bar{1} & 1 & 0 & 0 & \bar{1} & \bar{1} & \bar{1} & \bar{1}\\ 0 & \bar{1} & 0 & \bar{1} & \bar{1} & \bar{1} & \bar{1} & \bar{1} & 1\end{array}]$$

For this 9-dimensional system, there are 24309 possible combinations of variables and their derivatives candidates for providing full observability (see the Supplementary Section S2). Dealing with all these potential solutions is still afordable with our symbolic technique but it would take a rather long computational time (about 2 h). In order to reduce the number of combinations to test, we computed the linear out-strength ${\sigma }_{{\rm{out}}}^{{\rm{lin}}}(i)$ of the 9 variables which are

$${\sigma }_{{\rm{out}}}^{{\rm{lin}}}\mathrm{(2)}={\sigma }_{{\rm{out}}}^{{\rm{lin}}}\mathrm{(4)}={\sigma }_{{\rm{out}}}^{{\rm{lin}}}\mathrm{(5)}={\sigma }_{{\rm{out}}}^{{\rm{lin}}}\mathrm{(6)}=0$$

and

$${\sigma }_{{\rm{out}}}^{{\rm{lin}}}\mathrm{(1)}={\sigma }_{{\rm{out}}}^{{\rm{lin}}}\mathrm{(3)}={\sigma }_{{\rm{out}}}^{{\rm{lin}}}\mathrm{(7)}={\sigma }_{{\rm{out}}}^{{\rm{lin}}}\mathrm{(8)}={\sigma }_{{\rm{out}}}^{{\rm{lin}}}\mathrm{(9)}=1.$$

These values indicate that variables *x*_2_, *x*_4_, *x*_5_, *x*_6_ are necessarily to be included in the list of variables to be measured for obtaining a full observability since none of them linearly affect the dynamics of the rest. The other five are candidate variables to be removed from the measurements. Their respective in-strengths coming from the non measured variables are:

$${\sigma }_{{\rm{in}}}^{{\rm{nm}}}\mathrm{(1)}={\sigma }_{{\rm{in}}}^{{\rm{nm}}}\mathrm{(3)}=\mathrm{3,}\,{\sigma }_{{\rm{in}}}^{{\rm{nm}}}\mathrm{(7)}={\sigma }_{{\rm{in}}}^{{\rm{nm}}}\mathrm{(8)}=\mathrm{5,}\,\,{\rm{and}}\,\,{\sigma }_{{\rm{in}}}^{{\rm{nm}}}\mathrm{(9)}=4.$$

To determine whether all the candidate variables could be removed, we checked if there are pairs of exclusive variables, that is, when two candidate variables are linearly coupled each other (one being linearly “seen” by the other). Among the off-diagonal elements ${\tilde{J}}_{ij}$ equal to 1, we have ${\tilde{J}}_{17}={\tilde{J}}_{71}$, ${\tilde{J}}_{29}$, ${\tilde{J}}_{38}={\tilde{J}}_{83}$, and ${\tilde{J}}_{49}$. Variables (*x*_1_, *x*_7_) and (*x*_3_, *x*_8_) thus form two pairs of mutually exclusive variables. Variable *x*_9_ is the single one not involved in an exclusive pair and can be removed from the set of measured variables. To decide which variable from each pair can be safely removed, we check which variables have the largest in-strength from the candidate variables to be non measured. The comparison returns that variables *x*_7_ and *x*_8_ are the ones to be removed since ${\sigma }_{{\rm{in}}}^{{\rm{nl}}}\mathrm{(7)}={\sigma }_{{\rm{in}}}^{{\rm{nm}}}\mathrm{(8)}=5 > {\sigma }_{{\rm{in}}}^{{\rm{nm}}}\mathrm{(1)}={\sigma }_{{\rm{in}}}^{{\rm{nm}}}\mathrm{(3)}=3$.

The first test to assess the accuracy in selecting the minimal set of variables providing the highest observability consists in systematically investigating those combinations where all the variables are measured except one. The symbolic observability coefficients are reported in Table 1. In all cases providing full observability with just a single variable not being considered, the discarded measure matches one of the candidate variables *x*_1_, *x*_3_, *x*_7_, *x*_8_ and *x*_9_ (marked in bold face in the table) confirming our preselection analysis.

**Table 1:** All possible subsets with *m* = 8 measured variables and one Lie derivative (of the variable for which “2” is reported) providing a full observability of the state space associated with the 9-dimensional system (8). Those variables not affecting the full observability when not measured are highlighted in bold face.

*m*	*x*_1_	*x*_2_	*x*_3_	*x*_4_	*x*_5_	*x*_6_	*x*_7_	*x*_8_	*x*_9_	*η*
8	1	2	1	1	1	1	1	1	—	1.00
8	1	1	2	1	1	1	1	—	1	1.00
8	1	1	1	2	1	1	1	—	1	1.00
8	1	1	1	2	1	1	1	1	—	1.00
8	2	1	1	1	1	1	—	1	1	1.00
8	1	1	—	1	1	1	1	2	1	1.00
8	1	1	—	1	1	1	2	1	1	1.00
8	—	1	1	1	1	1	2	1	1	1.00

Our systematic computation of the symbolic coefficients allows us to quantify the number of times *N*_*i*_(*η*) the variable *x*_*i*_ is not part of an embedding providing a given observability value *η*. In particular, the values of *N*_*i*_(*η* = 1.0) for the variables potentially candidate to be non measured (*N*_1_(1.0) = 4, *N*_3_(1.0) = 2, *N*_7_(1.0) = 7, *N*_8_(1.0) = 5, and *N*_9_(1.0) = 9, see the first part of Table 2), support our initial choice for not measuring *x*_7_, *x*_8_, and *x*_9_ but measuring *x*_1_ and *x*_3_, since *x*_7_, *x*_8_ and *x*_9_ seem to be less essential for providing full observability. Consequently, as long as full observability is required, our two network-based criteria correctly identify those variables whose absence from the set of sensors does not affect the full observability of the system. Indeed, when the minimal number of variables, that is, *m* = 6, is measured, the two possible combinations providing a full observability correspond to a space reconstructed from variables *x*_1_, *x*_2_, *x*_3_, *x*_4_, *x*_5_ and *x*_6_ (see the Supplementary Table S1. We therefore correctly assessed the best variables to measure for getting full observability with the minimum of variables. Of course, it is also possible to get full observability by measuring more than 6 variables. In that case, we searched for them among the 8 preselected variables using the linear out-strength. From the 354 possible combinations, we obtained 6 combinations using 7 measured variables and 2 with 8 measured variables. Performing a full blind search, with no preselection, from a total number of 1080 combinations with 7 or 8 measured variables, we found 2 and 6 additional combinations providing full observability, respectively. All of them are reported in the Supplementary Table S1.

**Table 2:** List of the different possible combinations of measured variables and their Lie derivative orders providing a symbolic observability coefficient *η* ≥ 0.75 of the state space associated with the 9-dimensional system (8).

*m*	*x*_1_	*x*_2_	*x*_3_	*x*_4_	*x*_5_	*x*_6_	*x*_7_	*x*_8_	*x*_9_	*η*
6	2	2	2	1	1	1	—	—	—	1.00
6	2	2	2	1	1	1	—	—	—	1.00
7	1	2	2	1	1	1	1	—	—	1.00
7	1	2	—	1	1	1	1	2	—	1.00
7	—	1	1	2	1	1	2	1	—	1.00
7	—	1	1	2	1	1	2	1	—	1.00
7	—	1	1	1	1	1	2	1	1	1.00
7	—	1	—	1	1	1	2	2	1	1.00
7	2	2	1	1	1	1	—	1	—	1.00
7	2	1	2	2	1	1	—	—	—	1.00
7	2	1	2	1	1	1	—	—	1	1.00
7	2	1	1	2	1	1	—	1	—	1.00
7	2	1	1	1	1	1	—	1	1	1.00
5	2	3	2	1	1	—	—	—	—	0.90
5	2	1	2	3	1	—	—	—	—	0.90
6	3	2	1	1	1	1	—	—	—	0.90
6	3	1	2	1	1	1	—	—	—	0.90
6	3	1	1	2	1	1	—	—	—	0.90
6	2	3	1	1	1	1	—	—	—	0.90
6	2	3	1	1	1	—	—	1	—	0.90
6	2	1	3	1	1	1	—	—	—	0.90
6	2	1	1	3	1	1	—	—	—	0.90
6	2	1	1	3	1	—	—	1	—	0.90
6	1	3	2	1	1	1	—	—	—	0.90
6	1	3	2	1	1	—	1	—	—	0.90
6	1	3	—	1	1	—	1	1	—	0.90
6	1	2	3	1	1	1	—	—	—	0.90
6	1	2	—	1	1	—	1	3	—	0.90
6	1	1	3	2	1	1	—	—	—	0.90
6	1	1	2	3	1	1	—	—	—	0.90
6	1	1	2	3	1	—	1	—	—	0.90
6	—	1	1	3	1	—	2	1	—	0.90
6	—	1	1	2	1	—	3	1	—	0.90
6	—	1	—	1	1	—	3	2	1	0.90
6	—	1	—	1	1	—	2	3	1	0.90
6	—	1	—	1	1	—	2	3	1	0.90
5	4	2	1	1	1	—	—	—	—	0.80
5	4	1	2	1	1	—	—	—	—	0.80
5	4	1	1	2	1	—	—	—	—	0.80
5	2	1	4	1	1	—	—	—	—	0.80
5	1	2	4	1	1	—	—	—	—	0.80
5	1	1	4	2	1	—	—	—	—	0.80
6	4	1	1	1	1	—	—	1	—	0.80
6	4	1	1	1	1	—	—	—	1	0.80
6	1	1	4	1	1	—	1	—	—	0.80
6	1	1	4	1	1	—	—	—	1	0.80

**Figure 2:**
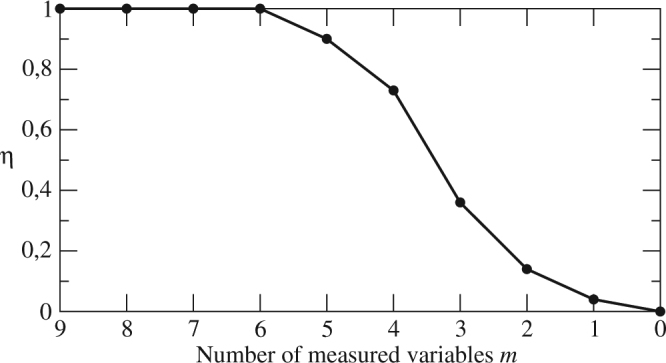
Largest symbolic observability coefficient *η versus* the number *m* of measured variables for the 9D Rayleigh-Bénard model (8).

We also investigated the combinations with less than 6 measured variables and providing the largest symbolic observability coefficients whose dependency on *m* is shown in Fig. 2. For *m* = 5 (*m* = 4, 3, 2 and 1) there are 4 (2, 6, 4, and 4, respectively) combinations with *η* = 0.90 (*η* = 0.73, 0.36, 0.14, 0.04, respectively). All those combinations are made up of the six preselected variables identified by solely using the symbolic Jacobian matrix and ${\sigma }_{{\rm{out}}}^{{\rm{lin}}}$ and ${\sigma }_{{\rm{in}}}^{{\rm{nm}}}$ to rank them.

The non-preselected variables can be involved in reconstructed vectors when a good but not a full observability is desired or when *m* > 6 (a good observability is considered when *η* > 0.75 as reported in a previous work^33^). For instance, this is exemplified in the middle and lower part of Table 2 with a systematic computation of the symbolic observability coefficients *η* for embeddings built from 5 or 6 measured variables. In this case, we observe that a very good observability (*η* = 0.90) can be obtained with only 5 variables being measured, namely (*x*_1_, *x*_2_, *x*_3_, *x*_4_, *x*_5_). The fact that variable *x*_6_ is not included, prevents from a full observability and, in particular, its absence induces a lack of symmetry, as previously discussed. Again, by looking at the distribution of *N*_*i*_(0.9) we have: *N*_1_(0.9) = 5, *N*_3_(0.9) = 5, *N*_7_(0.9) = 14, *N*_8_(0.9) = 14, and *N*_9_(0.9) = 20. Therefore, for this level of observability (*η* = 0.9) the last three variables, *x*_7_,*x*_8_, and *x*_9_ can be again chosen to be removed from the set of observations. If we accept a slightly lower observability coefficient (*η* = 0.80), other possibilities emerge in which variable *x*_6_ is systematicaly removed from the set of measured variables (last part of Table 2). Our two criteria are thus very efficient to detect those variables not really impacting the access to a full observability measure, but discard some possibilities offering a good (but not full until *m* ≤ 6) observability with severe consequences on the symmetry properties of the reconstructed attractor.

### A 13D model for DNA replication

A third and even more challenging case is now considered, a 13-dimensional model for the DNA replication in fission yeast. Fission yeast cells are carrying two mutant genes, wee1^−^ and cdc25^OP^, which initiate mitosis in eukaryotic cells before the end of their DNA replication. A second feature is that DNA synthesis can be restarted without intervening mitoses. Novak and Tyson proposed a model for cell cycle in fission yeast taking into account these two properties in *Schizosuccharomyces pombe*^34^. The underlying mechanisms are described by the set of thirteen differential equations

12 $$\{\begin{array}{rcl}{\dot{x}}_{1} & = & {k}_{1}-({k}_{2}+{k}_{{\rm{w}}ee}+{k}_{7}{x}_{2}){x}_{1}+{k}_{25}{x}_{8}+({k}_{7r}+{k}_{4}){x}_{4}\\ {\dot{x}}_{2} & = & {k}_{3}-{k}_{4}{x}_{2}-\frac{{k}_{{\rm{p}}}{x}_{2}({x}_{1}+\beta {x}_{8}+\alpha {x}_{3})m}{{K}_{{\rm{m}}p}+{x}_{2}}-{k}_{7}{x}_{2}({x}_{1}+{x}_{8})\\  &  & -\,{k}_{8}{x}_{2}{x}_{3}+({k}_{8r}+{k}_{6^{\prime} }){x}_{9}+({k}_{7r}+{k}_{2}+{k}_{2^{\prime} })({x}_{4}+{x}_{10})\\ {\dot{x}}_{3} & = & {k}_{5}-({k}_{6}+{k}_{8}{x}_{2}){x}_{3}+({k}_{8r}+{k}_{4}){x}_{9}\\ {\dot{x}}_{4} & = & {k}_{7}{x}_{2}{x}_{1}-({k}_{7r}+{k}_{4}+{k}_{2}+{k}_{2^{\prime} }){x}_{4}\\ {\dot{x}}_{5} & = & \frac{{k}_{{\rm{i}}}({x}_{1}+\beta {x}_{8}\mathrm{)(1}-{x}_{5})}{{K}_{{\rm{m}}i}+1-{x}_{5}}-\frac{{k}_{{\rm{i}}r}{x}_{5}}{{K}_{{\rm{m}}ir}+{x}_{5}}\\ {\dot{x}}_{6} & = & \frac{{k}_{u2}({x}_{1}+\beta {x}_{8}\mathrm{)(1}-{x}_{6})}{{K}_{{\rm{m}}u2}+1-{x}_{6}}-\frac{{k}_{{\rm{u}}r2}{x}_{6}}{{K}_{{\rm{m}}ur2}+{x}_{6}}\\ {\dot{x}}_{7} & = & \frac{{k}_{wr}\mathrm{(1}-{x}_{7})}{{K}_{{\rm{m}}wr}+1-{x}_{7}}-\frac{{k}_{{\rm{w}}}({x}_{1}+\beta {x}_{8}){x}_{7}}{{K}_{{\rm{m}}w}+{x}_{7}}\\ {\dot{x}}_{8} & = & {k}_{{\rm{w}}ee}{x}_{1}-({k}_{25}+{k}_{2}+{k}_{7}{x}_{2}){x}_{8}+({k}_{7r}+{k}_{4}){x}_{10}\\ {\dot{x}}_{9} & = & {k}_{8}{x}_{2}{x}_{3}-({k}_{8r}+{k}_{4}+{k}_{6^{\prime} }){x}_{9}\\ {\dot{x}}_{10} & = & {k}_{7}{x}_{2}{x}_{8}-({k}_{7r}+{k}_{4}+{k}_{2}+{k}_{2^{\prime} }){x}_{10}\\ {\dot{x}}_{11} & = & \frac{{k}_{{\rm{u}}}{x}_{5}\mathrm{(1}-{x}_{11})}{{K}_{{\rm{m}}u}+1-{x}_{11}}-\frac{{k}_{{\rm{u}}r}{x}_{11}}{{K}_{{\rm{m}}ur}+{x}_{11}}\\ {\dot{x}}_{12} & = & \frac{{k}_{{\rm{c}}}({x}_{1}+\beta {x}_{8}\mathrm{)(1}-{x}_{12})}{{K}_{{\rm{m}}c}+1-{x}_{12}}-\frac{{k}_{{\rm{c}}r}{x}_{12}}{{K}_{{\rm{m}}cr}+{x}_{12}}\\ \dot{m} & = & \mu m\end{array}$$

where *x*_1_ = G2K, *x*_2_ = R, *x*_3_ = G1K, *x*_4_ = G2R, *x*_5_ = IE, *x*_6_ = UbE2, *x*_7_ = Wee1, *x*_8_ = PG2, *x*_9_ = G1R, *x*_10_ = PG2R, *x*_11_ = UbE, and *x*_12_ = Cdc25, are concentration variables, see Novak and Tyson^34^ for a more detailed explanation of the meaning of these variables and values of the rate constants.

This 13-dimensional rational model is characterized by the symbolic Jacobian matrix

13 $${\mathop{J}\limits^{ \sim }}_{13{\rm{D}}}=[\begin{array}{ccccccccccccc}\bar{1} & \bar{1} & 0 & 1 & 0 & 0 & 0 & 1 & 0 & 0 & 0 & 0 & 0\\ \bar{1} & \bar{\bar{1}} & \bar{1} & 1 & 0 & 0 & 0 & \bar{1} & 1 & 1 & 0 & 0 & \bar{1}\\ 0 & \bar{1} & \bar{1} & 0 & 0 & 0 & 0 & 0 & 1 & 0 & 0 & 0 & 0\\ \bar{1} & \bar{1} & 0 & 1 & 0 & 0 & 0 & 0 & 0 & 0 & 0 & 0 & 0\\ \bar{1} & 0 & 0 & 0 & \bar{\bar{1}} & 0 & 0 & \bar{1} & 0 & 0 & 0 & 0 & 0\\ \bar{1} & 0 & 0 & 0 & 0 & \bar{\bar{1}} & 0 & \bar{1} & 0 & 0 & 0 & 0 & 0\\ \bar{1} & 0 & 0 & 0 & 0 & 0 & \bar{\bar{1}} & \bar{1} & 0 & 0 & 0 & 0 & 0\\ 1 & \bar{1} & 0 & 0 & 0 & 0 & 0 & \bar{1} & 0 & 1 & 0 & 0 & 0\\ 0 & \bar{1} & \bar{1} & 0 & 0 & 0 & 0 & 0 & 1 & 0 & 0 & 0 & 0\\ 0 & \bar{1} & 0 & 0 & 0 & 0 & 0 & \bar{1} & 0 & 1 & 0 & 0 & 0\\ 0 & 0 & 0 & 0 & \bar{1} & 0 & 0 & 0 & 0 & 0 & \bar{\bar{1}} & 0 & 0\\ \bar{1} & 0 & 0 & 0 & 0 & 0 & 0 & \bar{1} & 0 & 0 & 0 & \bar{\bar{1}} & 0\\ 0 & 0 & 0 & 0 & 0 & 0 & 0 & 0 & 0 & 0 & 0 & 0 & 1\end{array}]\,.$$

According to the linear out-strengths ${\sigma }_{{\rm{out}}}^{{\rm{lin}}}$, we have five candidate variables eligible to be excluded from the observations since having not null ${\sigma }_{{\rm{out}}}^{{\rm{lin}}}$ values:

$${\sigma }_{{\rm{out}}}^{{\rm{lin}}}\mathrm{(1)}={\sigma }_{{\rm{out}}}^{{\rm{lin}}}\mathrm{(8)}=1 < {\sigma }_{{\rm{out}}}^{{\rm{lin}}}\mathrm{(4)}={\sigma }_{{\rm{out}}}^{{\rm{lin}}}\mathrm{(9)}={\sigma }_{{\rm{out}}}^{{\rm{lin}}}\mathrm{(10)}=2.$$

The linear off-diagonal elements $({\tilde{J}}_{ij}=1)$ are ${\tilde{J}}_{14}$, ${\tilde{J}}_{18}$, ${\tilde{J}}_{24}$, ${\tilde{J}}_{29}$, ${\tilde{J}}_{\mathrm{2,10}}$, ${\tilde{J}}_{39}$, ${\tilde{J}}_{81}$, and ${\tilde{J}}_{\mathrm{8,10}}$. There is therefore a single pair of exclusive candidate variables, that is, variables *x*_1_ and *x*_8_ (${\tilde{J}}_{18}={\tilde{J}}_{81}\mathrm{=1}$). Variables *x*_4_, *x*_9_, and *x*_10_ can therefore be safely removed from the set of measured variables and *x*_1_ and *x*_8_ can not be simultaneously removed. The in-strength ${\sigma }_{{\rm{in}}}^{{\rm{nm}}}$ from the set of potentially non-measured variables is equal to 2 for all the candidate variables except for *x*_9_ which is ${\sigma }_{{\rm{in}}}^{{\rm{nm}}}\mathrm{(9)}=0$. Our criteria does not allow us this time to resolve the uncertainty between *x*_1_ and *x*_8_.

Following the same procedure as with the two previous examples, we performed our *a priori* analysis by systematically computing the symbolic observability coefficients when a single variable is removed and collecting only those combinations providing either full or null observability (see Table 3). As expected, when one of the variables *x*_1_, *x*_4_, *x*_8_, *x*_9_ or *x*_10_ is not included in the observation set of 12 variables plus one derivative, the observability is full. On the contrary, if one of the variables *x*_6_, *x*_7_, *x*_11_ and *x*_12_ is removed, the symbolic observability coefficient drops to zero for any possible choice of the first derivative. These variables are therefore essential and need to be measured. This is due to the fact that these variables have no out-connection other than to themselves as shown in the corresponding columns of the symbolic Jacobian matrix in Eq. (13).

**Table 3:** Symbolic observability coefficients when twelve (out of thirteen) variables of the DNA model (12) are measured. The derivative used for reconstructing a 13-dimensional state space is also reported.

Non-measured	Derivative retained	*η*
*x*_1_	${\dot{x}}_{8}$	1.00
*x*_4_	${\dot{x}}_{1}$ or ${\dot{x}}_{2}$	1.00
*x*_8_	${\dot{x}}_{1}$	1.00
*x*_9_	${\dot{x}}_{2}$ or ${\dot{x}}_{3}$	1.00
*x*_10_	${\dot{x}}_{2}$ or ${\dot{x}}_{8}$	1.00
*x*_6_	$\forall {\dot{x}}_{i}$	0.00
*x*_7_	$\forall {\dot{x}}_{i}$	0.00
*x*_11_	$\forall {\dot{x}}_{i}$	0.00
*x*_12_	$\forall {\dot{x}}_{i}$	0.00

**Figure 3:**
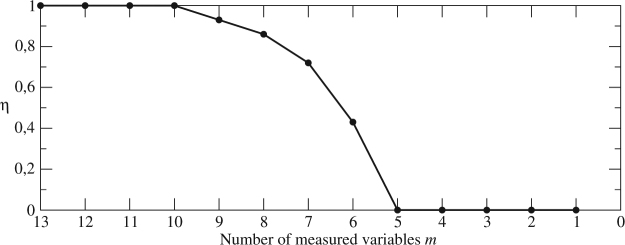
Largest symbolic observability coefficient *η versus* the number *m* of measured variables for the DNA model (12).

Let us now validate whether it is possible to retrieve a full observability when either the set {*x*_1_, *x*_4_, *x*_9_, *x*_10_} or {*x*_4_, *x*_8_, *x*_9_, *x*_10_} are removed from the list of variables to measure. This was performed by systematically computing the symbolic observability coefficients for all the combinations reconstructing a 13-dimensional space without taking into account those two sets of variables. We found that for this DNA model, there are not too many possibilities to reconstruct a space providing full observability of the original state space (see the Supplementary Table S1). For instance, when removing two of them, *x*_8_ and *x*_9_ the reconstructed state vector $({x}_{1},{\dot{x}}_{1},{x}_{2},{\dot{x}}_{2},{x}_{3},{\dot{x}}_{3},{x}_{4},{x}_{5},{x}_{6},{x}_{7},{x}_{11},{x}_{12},{x}_{13})$ is the only one providing full observability. If we discard three variables (*x*_8_, *x*_9_, and *x*_10_), there are two combinations allowing for a full observabiltiy embedding: $({x}_{1},{\dot{x}}_{1},{x}_{2},{\dot{x}}_{2},{x}_{3},{x}_{4},{x}_{5},{x}_{6},{x}_{7},{x}_{10},{x}_{11},{x}_{12},{x}_{13})$ and $({x}_{1},{\dot{x}}_{1},{x}_{2},{x}_{3},{\dot{x}}_{3},{x}_{4},{x}_{5},{x}_{6},{x}_{7},{x}_{10},{x}_{11},{x}_{12},{x}_{13})$. A systematic analysis of the symbolic observability coefficients as the number of variables are removed from the set of observations indicate that the coefficient already drops to 0.93 when *m* = 9 variables are measured (see Fig. 3). As the estimated threshold for an optimal observability is 0.75^33^, it is worthless to investigate sets of size smaller than *m* = 7.

To actually check the results accounted for the symbolic observability coefficients *η*_*s*_, we computed all the determinants Det ${\mathscr{O}}$_*s*_ corresponding to *η*_*s*_ = 1 (results are reported in the Supplementary Table S1). In the 14 cases for which the symbolic observability coefficients are equal to one, the determinant Det ${\mathscr{O}}$_*s*_ was always nonzero (for the whole state space): our technique always correctly identify reconstructed vectors providing full observability of the original space. As another example, as shown in Fig. 3, full observability is never achieved for *m* = 9 and the largest symbolic observability coefficient is 0.93, which still provides a good observability. By using the reconstructed space ${{\mathbb{R}}}^{13}({x}_{1},{\dot{x}}_{1},{x}_{2},{\dot{x}}_{2},{x}_{3},{\dot{x}}_{3},{x}_{5},{x}_{6},{x}_{7},{x}_{10},{x}_{11},{x}_{12},{\dot{x}}_{12},{x}_{13})$, (one of the cases reported in Table 4) the determinant

14 $${\rm{Det}}\,{\mathscr{O}}=-({k}_{{\rm{7}}r}+{k}_{2}+{k}_{{\rm{2p}}})\,({k}_{8{\rm{r}}}+{k}_{4})\,({k}_{{\rm{7r}}}+{k}_{4})\frac{{k}_{{\rm{c}}}\beta \,({x}_{12}-\mathrm{1)}}{{K}_{{\rm{mc}}}+1-{x}_{12}}$$

is zero for *x*_12_ = 1, a singular observability manifold of first order, explaining why the observability coefficient is no longer equal to 1 but close to it. To further show how the observability coefficient decreases when Det ${\mathscr{O}}$_*s*_ vanishes for a singularity of higher degree^22^, we computed the determinant

15 $${\rm{Det}}\,{{\mathscr{O}}}_{s}=-({k}_{{\rm{7r}}}+{k}_{2}+{k}_{{\rm{2p}}})\,({k}_{8{\rm{r}}}+{k}_{4})\,({k}_{{\rm{7r}}}+{k}_{4})\frac{{k}_{{\rm{u}}}\,{k}_{{\rm{c}}}\beta \,({x}_{11}-\mathrm{1)}\,({x}_{12}-\mathrm{1)}}{({K}_{{\rm{mc}}}+1-{x}_{12})\,({K}_{\mu }+1-{x}_{11})}$$

associated with the reconstructed space ${{\mathbb{R}}}^{13}({x}_{1},{\dot{x}}_{1},{x}_{2},{\dot{x}}_{2},{x}_{3},{\dot{x}}_{3},{x}_{6},{x}_{7},{x}_{10},{x}_{11},{\dot{x}}_{11},{x}_{12},{\dot{x}}_{12},{x}_{13})$ providing a slightly smaller observability (*η* = 0.86) in agreement with the singular observability manifold (15) of second order, defined by Det ${\mathscr{O}}$_*s*_ = 0, that is, by (*x*_11_ − 1)(*x*_12_ − 1) = 0. Due to a too large complexity, it was not possible to analytically compute the observability matrix when a single variable is measured.

**Table 4:** Symbolic observability coefficients for the DNA system. The first part corresponds to the case where variables {*x*_4_, *x*_8_, *x*_9_, *x*_10_} are not measured, the middle part to the case where variables {*x*_1_, *x*_4_, *x*_9_, *x*_10_} are not. Only the cases where the symbolic coefficient is non-zero and for which only a first derivative is used (to avoid too many possibilities) are reported.

*m*	*x*_1_	*x*_2_	*x*_3_	*x*_4_	*x*_5_	*x*_6_	*x*_7_	*x*_8_	*x*_9_	*x*_10_	*x*_11_	*x*_12_	*x*_13_	*η*
9	2	2	2	—	1	1	1	—	—	—	1	2	1	0.93
9	2	2	2	—	1	1	2	—	—	—	1	1	1	0.93
9	2	2	2	—	1	2	1	—	—	—	1	1	1	0.93
9	2	2	2	—	2	1	1	—	—	—	1	1	1	0.93
8	2	2	2	—	—	1	1	—	—	—	2	2	1	0.86
8	2	2	2	—	—	1	2	—	—	—	2	1	1	0.86
8	2	2	2	—	—	2	1	—	—	—	2	1	1	0.86
9	—	2	2	—	1	1	1	2	—	—	1	2	1	0.93
9	—	2	2	—	1	1	2	2	—	—	1	1	1	0.93
9	—	2	2	—	1	2	1	2	—	—	1	1	1	0.93
9	—	2	2	—	2	1	1	2	—	—	1	1	1	0.93
8	—	2	2	—	—	1	1	2	—	—	2	2	1	0.86
8	—	2	2	—	—	1	2	2	—	—	2	1	1	0.86
8	—	2	2	—	—	2	1	2	—	—	2	1	1	0.86
7	—	1	2	—	—	1	1	3	—	—	2	3	—	0.72
$\vdots$	$\vdots$	$\vdots$	$\vdots$	$\vdots$	$\vdots$	$\vdots$	$\vdots$	$\vdots$	$\vdots$	$\vdots$	$\vdots$	$\vdots$	$\vdots$	$\vdots$

As detailed in the Supplementary Section S1, Liu and coworkers’ graphical technique shows that by measuring the four variables *x*_6_, *x*_7_, *x*_11_, and *x*_12_ it is possible to estimate the states of the system. Clearly, our results are in strong disagreement. At this point, it is relevant to explain why our results are so different from those reported in previous works^16,17^. The first reason for the discrepancy is that Liu’s algorithm uses a linear theory, only taking into account whether the *i*th variable participates or not in the differential equation of variable *j* and not concerned on how is that dependence. The latter is just equivalent to use a symbolic Jacobian matrix equal to

16 $${\mathop{{\mathscr{J}}}\limits^{ \sim }}_{13{\rm{D}}}^{{\rm{l}}{\rm{i}}{\rm{n}}}=[\begin{array}{ccccccccccccc}1 & 1 & 0 & 1 & 0 & 0 & 0 & 1 & 0 & 0 & 0 & 0 & 0\\ 1 & 1 & 1 & 1 & 0 & 0 & 0 & 1 & 1 & 1 & 0 & 0 & 1\\ 0 & 1 & 1 & 0 & 0 & 0 & 0 & 0 & 1 & 0 & 0 & 0 & 0\\ 1 & 1 & 0 & 1 & 0 & 0 & 0 & 0 & 0 & 0 & 0 & 0 & 0\\ 1 & 0 & 0 & 0 & 1 & 0 & 0 & 1 & 0 & 0 & 0 & 0 & 0\\ 1 & 0 & 0 & 0 & 0 & 1 & 0 & 1 & 0 & 0 & 0 & 0 & 0\\ 1 & 0 & 0 & 0 & 0 & 0 & 1 & 1 & 0 & 0 & 0 & 0 & 0\\ 1 & 1 & 0 & 0 & 0 & 0 & 0 & 1 & 0 & 1 & 0 & 0 & 0\\ 0 & 1 & 1 & 0 & 0 & 0 & 0 & 0 & 1 & 0 & 0 & 0 & 0\\ 0 & 1 & 0 & 0 & 0 & 0 & 0 & 1 & 0 & 1 & 0 & 0 & 0\\ 0 & 0 & 0 & 0 & 1 & 0 & 0 & 0 & 0 & 0 & 1 & 0 & 0\\ 1 & 0 & 0 & 0 & 0 & 0 & 0 & 1 & 0 & 0 & 0 & 1 & 0\\ 0 & 0 & 0 & 0 & 0 & 0 & 0 & 0 & 0 & 0 & 0 & 0 & 1\end{array}]\,.$$

and not the one defined in Eq. (13). Despite there are more than 2100 combinations with 6 or 7 measured variables resulting in full observability, there is none with a single variable. However, with a linear approach, it is still possible to show that the combinations providing full observability correctly identify the variables which must necessarily be used (Table 5), that is, variables *x*_6_, *x*_7_, *x*_11_, and *x*_12_. Nevertheless, the observability is obviously overestimated (1 compared to 0.43 with a nonlinear theory) and, in addition, this approach does not allow to select what derivatives to use for spanning the reconstructed space. Assessing the observability of a network with a linear theory thus provides very poor and misleading results.

**Table 5:** Number *N* of combinations providing a full observability—according to a linear theory—when *m* variables are measured. The numbers *M*_*i*_ in which the *i*th variable is involved in a vector spanning the reconstructed state space providing a full observability are also reported. In bold, the four variables which are the most often involved.

*m*	*N*	*M*_1_	*M*_2_	*M*_3_	*M*_4_	*M*_5_	*M*_6_
6	230	9	87	111	62	0	**230**
7	1896	307	879	981	728	327	**1892**
*m*	*M*_7_	*M*_8_	*M*_9_	*M*_10_	*M*_11_	*M*_12_	*M*_13_
6	**230**	9	110	63	**230**	**230**	9
7	**1892**	303	978	722	**1891**	**1891**	470

## Discussion

The observability of a complex system refers to the property of being able to infer its whole state space by measuring the dynamics of a limited set of its variables. Determining the conditions that guarantee the full observability of a system involves testing a number of possibilities that increases exponentially with the dimension of that system and, for each case, it is required to compute the determinant of the observability matrix defining the singular observability manifold, that is, the subset of the state space that cannot be observed from the measurement^25^. It was shown in one of our previous works^2^ that for a five-dimensional rational system, the analytical computation of such a determinant may already exceed the capacity of softwares like Maple® or Mathematica®. Therefore, alternative approaches to investigate large complex systems are needed. Those proposed for instance by Sedoglavic^16^ or Liu and coworkers^17^ remain yet unsatisfactory as discussed by Wang and coworkers^35^, mainly because they do not provide a method to select which Lie derivatives accompany the measured variables and, more importantly, they do not consider a nonlinear observability theory appropriate to deal with nonlinear systems, nonlinearities occuring in the node dynamics or nonlinearly coupled units.

Actually, the treatment proposed by Sedoglavic^16^ is only probabilistic and tests local observability, not the global one. On the other hand, the graphical approach developed by Liu and co-workers^17^ is based on a linear description of the system which can only lead, by definition, to approximated results since, as previously discussed, the lack of observability mainly originates in the location (in the fluence graph) of nonlinear terms. We here investigated the three systems considered by Liu and coworkers (see the Supplementary Section S1) and showed that, in contrast with our results, theirs are not in agreement with the analytical predictions. In our previous work^2^, we investigated the same five-dimensional rational system considered by Sedoglavic^16^. While in the latter reference, the algorithm developed by the author identifies the first variable as the one providing (in fact local) observability, it is only poorly the case when the symbolic algorithm developed in the former one is applied to the possible combinations using this variable, even combined with other variables. And what is even more questionable, it is that when *x*_1_ is combined with one of the four other variables, *x*_2_, *x*_3_, *x*_4_ and *x*_5_, the largest symbolic observability coefficient is still very small, that is, 0.30, 0.18, 0.30, and 0.48, respectively.

We have shown how the efficiency of the algorithm initially proposed by Bianco-Martinez and coworkers^2^ is improved by identifying the minimal set of measured variables providing full observability before any search for the corresponding Lie derivatives. The reduced sets of candidate variables capable of fully reconstructing large reaction networks was correctly determined by analyzing the way the variables interact, by only applying two simple criteria on the symbolic Jacobian matrix of the networked system. For the 13 DNA model, there are 5.2 ⋅ 10^6^ possible combinations to test (see the Supplementary Section S2 for the details), requiring more than 18 days of computations with a 2.5 GHz Intel Core i5 processor. With our preselection of variables, only 2870 combinations are needed to be tested lowering the computation time to about 4 min, that is, by a factor greater than 1800! These criteria reduce drastically the time spent for searching candidate variables, thus providing the grounds to observe natural and man-made complex systems.

In order to evaluate the reliability of our procedure, we computed a success rate defined as the number of times a symbolic observability coefficient equal to 1 actually corresponds to getting a constant analytical determinant of the observability matrix. For the 42 combinations providing *η* = 1, the success rate is 100%. While we were able to identify and algebraically check all the resulting combinations for the 5D and 9D models, it was impossible for the 13D model due to the large amount of them. In the case of the 9D model, for which we obtained *m*_p_ = 6 preselected variables, our procedure missed 2 out of 8 combinations with *m* = 7, and 4 out of 6 with *m* = 8 (see the Supplementary Table S1). The missed combinations involve at least one variable which was not preselected and, consequently, not considered in our computations. When *m* = 6, some combinations are associated with a symbolic observability coefficient equal to 0.90: in that case, 36 out of 54 corresponding to this value of the symbolic observability coefficient were made up of the preselected variables. When *m* = 5 < *m*_p_, 100% of the combinations (34) associated with the largest symbolic observability coefficient (0.90) involved the preselected variables. It is important to note that, when *m* ≤ 6, all combinations providing the largest symbolic observability coefficient (see Fig. 2) are made up of the preselected variables (and are actually found). This means that the preselected variables are indeed the revelant ones for estimating the system states and, that all combinations using these variables are correctly identified. To the best of our knowledge, we have a single case for which the full observability was not detected by our procedure^33^: it corresponds to the rare case for which two nonlinear terms cancel each other in the computation of the determinant of the observability matrix.

Finally, as firstly reported by Parlitz and coworkers^36^, the observality of a system could be addressed by using delay coordinates. As shown by Gibson and coworkers^37^, delay coordinates are related to derivatives by a rotation and a rescaling. Consequently, any result valid for derivative coordinates (not affected by rotation and rescaling) holds for delay coordinates. The reduced sets of *m* measured variables (*m* < *d*) are not dependent on the use of delay or derivative coordinates, only on the choice of the complementary coordinates to reconstruct a *d*-dimensional space. Therefore, the extension of the technique proposed in this work to networks of discrete time systems (discretization of continuous-time systems) and iterated maps seems to be rather straightforward according, for instance, to Sarachik and Kreindler^38^ and to Nijmeijer^39^, respectively.

## Methods

### Introduction to observability theory

Our framework to quantify the observability of a dynamical system is here introduced with some definitions. Let us consider a *d*-dimensional dynamical system represented by the state vector *x* ∈ ${\mathbb{R}}$^*d*^ whose components are given by

17 $${\dot{x}}_{i}={f}_{i}({x}_{1},{x}_{2},{x}_{3},\ldots ,{x}_{d}),\,i=1,2,3,\ldots ,d$$

where *f*_*i*_ is the *i*th component of the vector field *f*.

Let us introduce the vector $\,s\in {{\mathbb{R}}}^{m}$ whose *m* components are the time series of measured variables given by the measurement function

18 $$\,s=\,h(x\mathrm{).}$$

One of the formal ways to define the observability of a system is as follows^40^. We provide such a definition in the case where a single scalar time series is measured, *s* = *h*(*x*), but a generalization to the case of *m* measured variables is straighforward. The dynamical system (17) is said to be *state observable* at time *t*_*f*_ if every initial state *x*(0) can be uniquely determined from the knowledge of a finite time series of the measured variable *s*(*τ*), 0 ≤ *τ* ≤ *t*_*f*_. In practice, it is possible to test whether the dynamical system (17) is observable through a measurement function by computing the rank of the observability matrix^20^, that is, the Jacobian matrix of the Lie derivatives of *s*. Differentiating *s*(*t*) yields

$$\dot{s}(t)=\frac{{\rm{d}}}{{\rm{d}}t}h({\boldsymbol{x}})=\frac{\partial h}{\partial {\boldsymbol{x}}\,}\dot{{\boldsymbol{x}}}=\frac{\partial h}{\partial {\boldsymbol{x}}}{\boldsymbol{f}}({\boldsymbol{x}})={ {\mathcal L} }_{f}h({\boldsymbol{x}}),$$

where ${\mathcal L}$_*f* _*h*(*x*) is the Lie derivative of *h* along the vector field *f*. The *j*th order Lie derivative is given by

$${ {\mathcal L} }_{f}^{j}h({\boldsymbol{x}})=\frac{\partial { {\mathcal L} }_{f}^{j-1}h(x)}{\partial {\boldsymbol{x}}}{\boldsymbol{f}}({\boldsymbol{x}}),$$

being the zero order Lie derivative the measured variable itself, ${ {\mathcal L} }_{f}^{0}h({\boldsymbol{x}})=h({\boldsymbol{x}})$. Therefore, the observability matrix ${{\mathscr{O}}}_{s}\in {{\mathbb{R}}}^{d\times d}$ can be written as

19 $${{\mathscr{O}}}_{s}({\boldsymbol{x}})=[\begin{array}{c}{\rm{d}}h({\boldsymbol{x}})\\ {\rm{d}}{{\mathscr{L}}}_{f}h({\boldsymbol{x}})\\ \vdots \\ {\rm{d}}{{\mathscr{L}}}_{f}^{d-1}h({\boldsymbol{x}})\end{array}]$$

where ${\rm{d}}\equiv \frac{\partial }{\partial {\boldsymbol{x}}}$ and the dynamical system (17) is said to be state observable if and only if the observability matrix has full rank, that is, rank (${\mathscr{O}}$_*s*_) = *d*. Notice that, the full observability of a system is determined by the space spanned not only by the measured variables but also by their appropriate Lie derivatives^1^.

The observability matrix ${\mathscr{O}}$_*s*_ corresponds in fact to the Jacobian matrix of the change of coordinates Φ_*s*_: ***x*** → ***X*** where ${\boldsymbol{X}}\in {{\mathbb{R}}}^{d}$ is the reconstructed state vector from the *m* measured variables and their adequately chosen *d* − *m* Lie derivatives^30^. Let us make explicit the observability matrix ${{\mathscr{O}}}_{{x}_{i}{x}_{j}}$ for the situation where two arbitrary variables *x*_*i*_ and *x*_*j*_ are measured directly, that is, when *s* = (*h*_1_(*x*),*h*_2_(*x*)) = (*x*_*i*_, *x*_*j*_), and *h*_1_ and *h*_2_ are two measurement functions. In this case, the observability matrix reads as,

20 $${{\mathscr{O}}}_{{x}_{i}{x}_{j}}=[\begin{array}{c}{\rm{d}}{h}_{1}({\boldsymbol{x}})\\ {\rm{d}}{{\mathscr{L}}}_{{f}_{i}}{h}_{1}({\boldsymbol{x}})\\ \vdots \\ {\rm{d}}{{\mathscr{L}}}_{{f}_{i}}^{k}{h}_{1}({\boldsymbol{x}})\\ {\rm{d}}{h}_{2}({\boldsymbol{x}})\\ {\rm{d}}{{\mathscr{L}}}_{{f}_{j}}{h}_{2}({\boldsymbol{x}})\\ \vdots \\ {\rm{d}}{{\mathscr{L}}}_{{f}_{j}}^{l}{h}_{2}({\boldsymbol{x}})\end{array}]$$

where the order Lie derivatives *k* and *l* are such that *k* + *l* = *d* − 2, that is, there are *d* − 2 + 1 possibilities for choosing *k* and *l*. According to the Takens theorem^41^, it is possible to increase the dimension up to 2*d*_H_ + 1 where *d*_H_ is ideally the Haussdorff dimension of the attractor to ensure a global diffeomorphism between the original state space and the reconstructed one, as long as the measurement function is generic. Showing that a measurement function is generic is not a trivial problem which is out of the scope of the present work. There is, therefore, no guarantee that the Takens theorem applies here. Moreover, our aim is to select the minimal set of measurements providing the best observability of the system. When a higher-dimensional reconstructed space is considered, this means that a global diffeomorphism perhaps may be obtained but it also means that the measurements provide information that is non-optimal and from which the analysis is most likely problematic and tricky^42,43^. Consequently, investigating higher-dimensional reconstructed spaces has a rather limited interest in the present context.

The fact the system is fully observable from the two measured variables considered in the matrix (20) depends also on the particular choice of the pair (*k*, *l*), the numbers of successive derivatives computed from *x*_*i*_ and *x*_*j*_, respectively. Therefore, it is crucial to specify how the measured variables and their derivatives are used to reconstruct the state space. An approach–as the ones developed in other works^16–18^–missing this necessary condition cannot indeed properly address the problem of full (or even good) observability.

### Symbolic observability formalism

The procedure to calculate the symbolic observability coefficients is implemented in four steps as follows:

i) *Construction of the symbolic Jacobian matrix* ($\tilde{{\mathscr{J}}}$). The Jacobian matrix ${\mathscr{J}}$, composed of elements *J*_*ij*_, of the system (17) is transformed into a symbolic Jacobian matrix $\tilde{{\mathscr{J}}}$ by replacing each linear element *J*_*ij*_ by 1, each non-linear polynomial element *J*_*ij*_ by, and each rational element *J*_*ij*_ by when the *j* th variable is present in the denominator or by $\overline{1}$ otherwise. This is more or less equivalent to the so-called influence (or fluence) diagram as used by Letellier and Aguirre^23^ where linear and nonlinear coupling terms are associated with solid and dashed arrows, respectively, and as used by Liu and coworkers^17^ where coupling terms are labelled with arrows (without distinguishing linear from nonlinear couplings). In the present approach, rational terms are distinguished from nonlinear polynomial terms since they strongly reduce the observability^2^.

ii) *Construction of the symbolic observability matrix* ($\tilde{O}$_*s*_). Let us consider for simplicity a univariate measurement *s* = *h*(*x*) = *x*_*i*_. For this particular case, the first row of $\tilde{O}$_*s*_ is just defined by the derivative of the measurement function d*h*, that is, ${\tilde{O}}_{1j}\mathrm{=1}$ if *j* = *i* and 0 otherwise. The second row is directly obtained from $\tilde{{\mathscr{J}}}$ by copying its *i*th row, that is, ${\tilde{O}}_{2j}={\tilde{J}}_{ij}$ ∀*j*, being *i* the index of the measured variable. The *k*th row is obtained as follows. First, each element of the *i*th row of the symbolic Jacobian observability matrix $\tilde{{\mathscr{J}}}$ is multiplied by the corresponding *i*th component of the vector $v=({\tilde{O}}_{\ell 1},\cdots ,{\tilde{O}}_{\ell d})$ where $\ell =k-1$ refers to the (*k* − 1)th row of the symbolic observability matrix $\tilde{O}$_*s*_. The rules to perform the symbolic product ${\tilde{J}}_{ij}\otimes {v}_{i}$ are such that^2^

21 $$|\begin{array}{ccc}0\otimes a & = & 0,\\ 1\otimes a & = & a,\\ \bar{1}\otimes a & = & a\,{\rm{f}}{\rm{o}}{\rm{r}}\,a=\bar{1},\bar{\bar{1}},\\ \bar{\bar{1}}\otimes a & = & \bar{\bar{1}}\,{\rm{f}}{\rm{o}}{\rm{r}}\,a\ne 0.\end{array}$$

Second, the resulting symbolic Jacobian matrix $\tilde{{\mathscr{J}}}$′ is thus reduced into a row where each element ${\tilde{O}}_{kj}={\sum }_{i}\tilde{J}{\text{'}}_{ij}$ is just the sum of the elements of the *j*th column according to the addition law^2^

22 $$|\begin{array}{ccc}0\oplus a & = & a,\\ 1\oplus a & = & a\,{\rm{f}}{\rm{o}}{\rm{r}}\,a\ne 0,\\ \bar{1}\oplus a & = & a\,{\rm{f}}{\rm{o}}{\rm{r}}\,a\ne 0,\,1,\\ \bar{\bar{1}}\oplus a & = & \bar{\bar{1}}.\end{array}$$

In the case *m* variables are measured, the construction of $\tilde{O}$_***s***_ is performed by blocks of size (*d*_*i*_ + 1) × *d*, being *d*_*i*_ the number of derivatives of *s*_*i*_ and ${\sum }_{i\mathrm{=1}}^{m}{d}_{i}+m=d$, and the construction of each block follows the same rules described above for univariate measures.

iii) *Computation of the symbolic observability coefficients*. The determinant of $\tilde{O}$_***s***_ is computed according to the symbolic product rule defined in (21) and expressed as products and addends of the symbolic terms 1, $\overline{1}$ and $\overline{\overline{1}}$, whose number of occurrences are stored in variables *N*_1_, *N* and *N*, respectively. A special condition is required for rational systems such that, if *N* = 0 and *N* ≠ 0 then *N* = *N*. The symbolic observability coefficient for the measurement ***s*** is then equal to

23 $${\eta }_{{\boldsymbol{s}}}=\frac{1}{D}{N}_{1}+\frac{1}{{D}^{2}}{N}_{\overline{1}}+\frac{1}{{D}^{3}}{N}_{\overline{\overline{1}}}$$

with *D* = max(1, *N*_1_) + *N* + *N* and 0 ≤ *η*_***s***_ ≤ 1, being *η*_***s***_ = 1 for a combination providing full observability.

iv) *Selecting the miminal set of variables to measure and the adequate Lie derivatives for providing a full observability*. The symbolic observability coefficients *η*_***s***_ for each one of the sets of *m* measured variables and their selected *d* − *m* Lie derivatives are ranked versus the decreasing value of *η*_***s***_ and increasing *m*. Those featuring *η*_***s***_ = 1 and the smallest *m* can be selected as the minimal sets of variables to measure.

## Electronic supplementary material

Supplementary material
