# Supplementary material for: A symbolic network-based nonlinear theory for dynamical systems observability
Source: Sci Rep. 2018 Feb 28;8:3785. doi: 10.1038/s41598-018-21967-w (PMC5830642; doi:10.1038/s41598-018-21967-w)
Supplement: Supplementary file 1 — Supplementary material [file 41598_2018_21967_MOESM1_ESM.pdf]

A symbolic network-based nonlinear theory for dynamical systems observability

Supplementary Materials

Christophe Letellier[1,*], **Irene Sendiña-Nadal**[2,3], **Ezequiel Bianco-Martinez**[4], **and Murilo S. Baptista**[4]

[1]CORIA-UMR 6614 Normandie Université, Campus Universitaire du Madrillet, F-76800 Saint-Etienne du Rouvray, France
[2]Complex Systems Group, Universidad Rey Juan Carlos, 28933 Móstoles, Madrid, Spain
[3]Center for Biomedical Technology, Universidad Politécnica de Madrid, 28223 Pozuelo de Alarcón, Madrid, Spain
[4]Institute for Complex Systems and Mathematical Biology, SUPA, University of Aberdeen, Old Aberdeen AB24 3UE United Kingdom
[*]Christophe.Letellier@coria.fr

ABSTRACT

This supplementary materials provides a detailed comparison between the symbolic approach we developed and the procedure based on inference graphs as introduced by Liu and coworkers'. The number of possible combinations are details and all combinations providing a full observability were checked by an analytical computation of the determinant of the corresponding observability matrix.

1 Comparison with the approach using inference diagram

In Liu and coworkers' approach,[1] the first step is to consider the so-called inference diagram by drawing an oriented link $x_i \to x_j$ when x_j occurs in the ith governing equation ($\dot{x}_i$) of the system. Links (auto-loops) $x_i \to x_i$ are discarded since they do not contribute to the connectivity of the network. Then the inference diagram is decomposed into strongly connected components (SCC) in such a way that any pair of nodes belonging to a given SCC are connected *via* an oriented path. When there is no incoming links from a node outside of a given SCC, such SCC is a *root* SCC, as the ones encircled by dashed lines in Figs. 1-4. It is then assumed that it is sufficient to measure one variable in each root SCC for estimating the states of the network.

For instance, the Rössler system[2]

$$\left\{ \begin{array}{l} \dot{x} = -y - z \\ \dot{y} = x + ay \\ \dot{z} = b + z(x - c) \end{array} \right. \tag{1}$$

is characterized by the inference diagram shown in Fig. 1. There is a single root SCC. It should be therefore sufficient to measure one of the three variables to estimate the states of the system. However, this is not correct since the symbolic observability coefficients are equal to $\eta_{x^3} = 0.88$, $\eta_{y^3} = 1$, and $\eta_{z^3} = 0.44$, respectively.[3] Rigorously speaking only variable y provides a full observability. When variable x or z is measured, there is always a domain of the original state space which is not observable,[4] meaning that, the states of that domain cannot be estimated. Consequently, the observability of the original dynamics strongly depends on the variable measured.[3] The inference diagram does not allow to discriminate the observability of the original state space provided by these different variables.

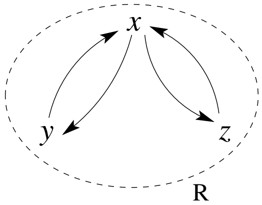

Figure 1. Inference diagram of the Rössler system (1). Root SCCs are encircled with dashed lines and labeled with R.

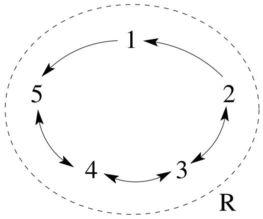

Figure 2. Inference diagram of the 5D rational model (2). Root SCCs are encircled with dashed lines and labeled with R.

The inference diagram of the 5D rational model[5]

$$\left\{\begin{array}{l}\dot{x}_1 = \dfrac{v_s K_I^4}{K_I^4 + x_5^4} - \dfrac{v_m x_1}{K_m + x_1} \\ \dot{x}_2 = k_s x_1 - \dfrac{V_1 x_2}{K_1 + x_2} + \dfrac{V_2 x_3}{K_2 + x_3} \\ \dot{x}_3 = \dfrac{V_1 x_2}{K_1 + x_2} + \dfrac{V_4 x_4}{K_4 + x_4} - x_3 \left(\dfrac{V_2}{K_2 + x_3} + \dfrac{V_3}{K_3 + x_3} \right) \\ \dot{x}_4 = \dfrac{V_3 x_3}{K_3 + x_3} - x_4 \left(\dfrac{V_4}{K_4 + x_4} + k_1 + \dfrac{v_d}{K_d + x_4} \right) + k_2 x_5 \\ \dot{x}_5 = k_1 x_4 - k_2 x_5 \end{array}\right. \tag{2}$$

is shown in Fig. 2. There is a single root SCC. Thus, according to this approach, measuring one of the variables of the system should be sufficient to estimate the states of the system. This is in strong contradiction with our results since, for instance when variable x_3 is measured, the symbolic observability coefficient is $\eta_{x_3^5} = 0.02$, thus corresponding to an extremely poor observability. The situation when variables x_2 or x_4 are measured is only slightly better since $\eta_{x_2^5} = 0.08$, and $\eta_{x_4^5} = 0.09$, respectively (note that here observability is considered good[8] when $\eta > 0.75$). Our procedure shows instead that at least three variabels must be measured for having a full observability of the original state space.

The inference diagram of the 9D Rayleigh-Bénard model[6]

$$\left\{\begin{array}{ll}\dot{x}_1 = & -\sigma b_1 x_1 - x_2 x_4 + b_4 x_4^2 + b_3 x_3 x_5 - \sigma b_2 x_7 \\ \dot{x}_2 = & -\sigma x_2 + x_1 x_4 - x_2 x_5 + x_4 x_5 - \sigma x_9/2 \\ \dot{x}_3 = & -\sigma b_1 x_3 + x_2 x_4 - b_4 x_2^2 - b_3 x_1 x_5 + \sigma b_2 x_8 \\ \dot{x}_4 = & -\sigma x_4 - x_2 x_3 - x_2 x_5 + x_4 x_5 + \sigma x_9/2 \\ \dot{x}_5 = & -\sigma b_5 x_5 + x_2^2/2 - x_4^2/2 \\ \dot{x}_6 = & -b_6 x_6 + x_2 x_9 - x_4 x_9 \\ \dot{x}_7 = & -b_1 x_7 - R x_1 + 2 x_5 x_8 - x_4 x_9 \\ \dot{x}_8 = & -b_1 x_8 + R x_3 - 2 x_5 x_7 + x_2 x_9 \\ \dot{x}_9 = & -x_9 - R x_2 + R x_4 - 2 x_2 x_6 + 2 x_4 x_6 + x_4 x_7 - x_2 x_8 \end{array}\right. \tag{3}$$

is shown in Fig. 3. The high connectivity of this reaction network is such that there is again a single root SCC. Consequently, according to the inference diagram, any variable of the system should provide a correct estimation of the system states. Nevertheless, the observability matrix is rank deficient when variables x_5, x_6, or x_7 are measured and the observability is extremely poor when only of the other variables are measured ($\eta_{x_2^9} = \eta_{x_4^9} = 0.03$ and $\eta_{x_1^9} = \eta_{x_3^9} = \eta_{x_7^9} = \eta_{x_8^9} = 0.04$). Again, the inference diagram does not provide a reliable information. We showed that at least six variables must be measured for having full observability of this 9D model.

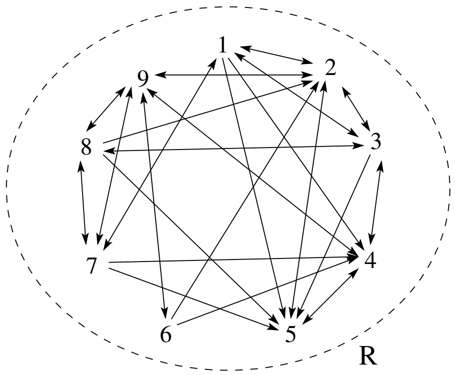

Figure 3. Inference diagram of the 9D Rayleigh-Bénard model (3). Root SCCs are encircled with dashed lines and labeled with R.

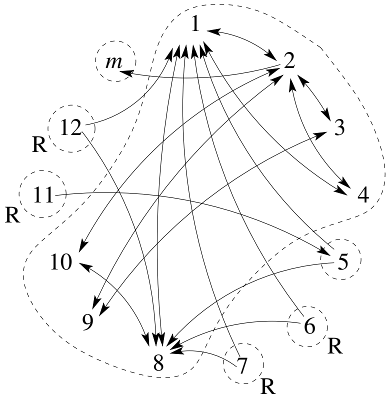

Figure 4. Inference diagram of the 13D DNA model (4). Root SCCs are encircled with dashed lines and labeled with R.

The inference diagram of the 13D DNA model[7]

$$
\left\{
\begin{array}{ll}
\dot{x}_1 & = k_1 - (k_2 + k_{\rm wee} + k_7 x_2) x_1 + k_{25} x_8 + (k_{7r} + k_4) x_4 \\
\dot{x}_2 & = k_3 - k_4 x_2 - \dfrac{k_{\rm p} x_2 (x_1 + \beta x_8 + \alpha x_3) m}{K_{\rm mp} + x_2} - k_7 x_2 (x_1 + x_8) - k_8 x_2 x_3 + (k_{8r} + k_{6'}) x_9 \\
 & + (k_{7r} + k_2 + k_{2'})(x_4 + x_{10}) \\
\dot{x}_3 & = k_5 - (k_6 + k_8 x_2) x_3 + (k_{8r} + k_4) x_9 \\
\dot{x}_4 & = k_7 x_2 x_1 - (k_{7r} + k_4 + k_2 + k_{2'}) x_4 \\
\dot{x}_5 & = \dfrac{k_{\rm i}(x_1 + \beta x_8)(1 - x_5)}{K_{\rm mi} + 1 - x_5} - \dfrac{k_{\rm ir} x_5}{K_{\rm mir} + x_5} \\
\dot{x}_6 & = \dfrac{k_{u2}(x_1 + \beta x_8)(1 - x_6)}{K_{\rm mu2} + 1 - x_6} - \dfrac{k_{\rm ur2} x_6}{K_{\rm mur2} + x_6} \\
\dot{x}_7 & = \dfrac{k_{wr}(1 - x_7)}{K_{\rm mwr} + 1 - x_7} - \dfrac{k_{\rm w}(x_1 + \beta x_8) x_7}{K_{\rm mw} + x_7} \\
\dot{x}_8 & = k_{\rm wee} x_1 - (k_{25} + k_2 + k_7 x_2) x_8 + (k_{7r} + k_4) x_{10} \\
\dot{x}_9 & = k_8 x_2 x_3 - (k_{8r} + k_4 + k_{6'}) x_9 \\
\dot{x}_{10} & = k_7 x_2 x_8 - (k_{7r} + k_4 + k_2 + k_{2'}) x_{10} \\
\dot{x}_{11} & = \dfrac{k_{\rm u} x_5 (1 - x_{11})}{K_{\rm mu} + 1 - x_{11}} - \dfrac{k_{\rm ur} x_{11}}{K_{\rm mur} + x_{11}} \\
\dot{x}_{12} & = \dfrac{k_{\rm c}(x_1 + \beta x_8)(1 - x_{12})}{K_{\rm mc} + 1 - x_{12}} - \dfrac{k_{\rm cr} x_{12}}{K_{\rm mcr} + x_{12}} \\
\dot{m} & = \mu m
\end{array}
\right. \qquad (4)
$$

is shown in Fig. 4. There are seven SCC, among which four are root SCCs. According to the inference diagram, measuring simultaneously variables x_6, x_7, x_{11} and x_{12} should be sufficient to estimate the states of the 13D DNA model. This is not confirmed by our results since the observability matrix obtained with four measured variables is always rank deficient. The inference diagram does not accurately assess the observability of this reaction network.

2 Number of combinations

For a d-dimensional system for which m variables are measured, the number of different vectors which can span the reconstructed space $\mathbb{R}^d$ is given by

$$
N_c = \sum_{m=1}^{N-1} \left(\begin{array}{c} d \\ m \end{array} \right) \cdot \left(\left(\begin{array}{c} d-m \\ m \end{array} \right) \right) \text{ where } \left(\begin{array}{c} d \\ m \end{array} \right) = \frac{n!}{k!(n-k)!} \qquad (5)
$$

is the binomial coefficient providing the number of ways to choose a subset from a set of elements, and $\left(\!\!\binom{n}{k}\!\!\right) = \binom{n+k-1}{k}$ provides the number of ways to choose a subset of k elements from a set of n elements which can be repeatedly selected.

For a d-dimensional system for which m variables are measured and $m_\text{p} \leq m$ of them are preselected, and for which there are δ pairs of exclusive variables (at least one of each pair must be measured), the number of possible combinations is

$$N'_c = \sum_{k=0}^{\delta} 2^{\delta-k} \binom{\delta}{k} \cdot \left(\!\!\binom{m_\text{p}+k}{d-(m_\text{p}+k)}\!\!\right) + \sum_{k=\delta+1}^{d-1} \binom{m_\text{d}}{k-\delta} \cdot \left(\!\!\binom{m_\text{p}+k}{m_\text{d}}\!\!\right) \quad (6)$$

where $m_\text{d} = d - m - 2\delta + k - 1$.

3 Success rate

References

1. Liu, Y.-Y., Slotine, J.-J. & Barabási, A.-L., Observability of complex systems, *Proc. Natl. Acad. Sci.* (USA), **110**, 2460-2465 (2013).
2. Rössler, O. E., An equation for continuous chaos, *Phys. Lett. A* **57**, 397 (1976).
3. Bianco-Martinez, E., Baptista, M. S., & Letellier, C., Symbolic computations of non-linear observability, *Phys. Rev. E* **91**, 062912 (2015).
4. Frunzete, M., Barbot, J.-P. & Letellier, C., Influence of the singular manifold of non-observable states in reconstructing chaotic attractors, *Phys. Rev. E* **86**, 026205 (2012).
5. Goldbeter, A., A model for circadian oscillations in the *Drosophila* period protein, *Proc. Royal Soc. B* **261**, 319-324, (1995).
6. Reiterer, P., Lainscsek, C., Schürrer, F., Letellier, C. & Maquet, J., A nine-dimensional Lorenz system to study high-dimensional chaos, *J. Phys. A* **31**, 7121-7139 (1998).
7. Novak, B. & Tyson, J. J., Modeling the control of DNA replication in fission yeast, *Proc. Natl. Acad. Sci.* **94**, 9147-9152 (1997).
8. Sendiña-Nadal, I., Boccaletti, S. & Letellier, C., Observability coefficients for predicting the class of synchronizability from the algebraic structure of the local oscillators, *Phys. Rev. E* **94**, 042205 (2016).

Table 1. Symbolic observability coefficients η and the corresponding analytical determinant of the observability matrix Det $\mathscr{O}$ for the 3D Rössler system, the 5D Drosophila model, the 9D Rayleigh-Bénard model and, the 13 DNA model. The success rate for these systems is 100%. The number m of measured variables is also reported.

m	Observability coeff.	Det $\mathscr{O}$	m	Observability coeff.	Det $\mathscr{O}$
3D Rössler system			5D Drosophila model		
1	$\eta_{y^3} = 1$	1	3	$\eta_{x_2^2 x_3 x_4^2} = 1$	$-k_s k_2$
2	$\eta_{x^2 y} = 1$	1	3	$\eta_{x_2^2 x_3 x_5^2} = 1$	$k_1 k_s$
2	$\eta_{x^2 z} = 1$	-1	4	$\eta_{x_1 x_2 x_3 x_4^2} = 1$	k_2
2	$\eta_{y^2 z} = 1$	-1	4	$\eta_{x_1 x_2 x_3 x_5^2} = 1$	$-k_1$
9D Rayleigh-Bénard model			4	$\eta_{x_2^2 x_3 x_4 x_5} = 1$	$-k_s$
6	$\eta_{x_1^2 x_2^2 x_3^2 x_4 x_5 x_6} = 1$	$-\frac{b_2^2 \sigma^3}{2}$	4	$\eta_{x_2^2 x_3 x_4 x_5} = 1$	$-k_s$
6	$\eta_{x_1^2 x_2 x_3^2 x_4^2 x_5 x_6} = 1$	$-\frac{b_2^2 \sigma^3}{2}$	2	$\eta_{x_2^2 x_5^3} = 0.70$	$k_1^2 k_s \left[\frac{V_3}{K_3+x_3} - \frac{V_3 x_3}{(K_3+x_3)^2}\right]$
7	$\eta_{x_2 x_4 x_5 x_6 x_7^2 x_8^2 x_9} = 1$	$-R^2$	13D DNA model		
7	$\eta_{x_2 x_3 x_4^2 x_5 x_6 x_7^2 x_8} = 1$	$\frac{R\sigma}{2}$	10	$\eta_{x_1^2 x_2^2 x_3^2 x_4 x_5 x_6 x_7 x_{11} x_{12} x_{13}} = 1$	$(k_{7r} + k_2 + k_{2'})(k_{8r} + k_4) k_{25}$
7	$\eta_{x_1 x_2 x_3^2 x_4^2 x_5 x_6 x_7} = 1$	$-\frac{b_2 \sigma^2}{2}$	10	$\eta_{x_1 x_2^2 x_3^2 x_5 x_6 x_7 x_8^2 x_{11} x_{12} x_{13}} = 1$	$-(k_{7r} + k_2 + k_{2'})(k_{8r} + k_4)(k_{7r} + k_4)$
7	$\eta_{x_1 x_2^2 x_4 x_5 x_6 x_7 x_8^2} = 1$	$\frac{R\sigma}{2}$	10	$\eta_{x_1^2 x_2 x_3^2 x_5 x_6 x_7 x_8^2 x_{11} x_{12} x_{13}} = 1$	$(k_{7r} + k_4)^2 (k_{8r} + k_4)$
7	$\eta_{x_1 x_2^2 x_3^2 x_4 x_5 x_6 x_7} = 1$	$-\frac{b_2 \sigma^2}{2}$	10	$\eta_{x_1^2 x_2^2 x_3 x_5 x_6 x_7 x_8^2 x_{11} x_{12} x_{13}} = 1$	$-(k_{7r} + k_4)^2 (k_{8r} + k_{6'})$
7	$\eta_{x_1^2 x_2 x_3 x_4^2 x_5 x_6 x_8} = 1$	$-\frac{b_2 \sigma^2}{2}$	10	$\eta_{x_2^2 x_3^2 x_5 x_6 x_7 x_8^2 x_{10} x_{11} x_{12} x_{13}} = 1$	$-k_{\mathrm{wee}}(k_{7r} + k_2 + k_{2'})(k_{8r} + k_4)$
7	$\eta_{x_1^2 x_2 x_3^2 x_4 x_5 x_6 x_9} = 1$	$-\frac{b_2 \sigma^2}{2}$	10	$\eta_{x_1^2 x_2^2 x_3^2 x_5 x_6 x_7 x_8 x_{11} x_{12} x_{13}} = 1$	$(k_{7r} + k_2 + k_{2'})(k_{8r} + k_4)(k_{7r} + k_4)$
7	$\eta_{x_1^2 x_2^2 x_3 x_4 x_5 x_6 x_8} = 1$	$\frac{b_2 \sigma^2}{2}$	11	$\eta_{x_1^2 x_2 x_3^2 x_4 x_5 x_6 x_7 x_{10} x_{11} x_{12} x_{13}} = 1$	$(k_{8r} + k_4) k_{25}$
8	$\eta_{x_1 x_2 x_4 x_5 x_6 x_7 x_8^2 x_9} = 1$	$-R$	11	$\eta_{x_1^2 x_2^2 x_3 x_4 x_5 x_6 x_7 x_{10} x_{11} x_{12} x_{13}} = 1$	$-(k_{8r} + k_{6'}) k_{25}$
8	$\eta_{x_2 x_3 x_4 x_5 x_6 x_7^2 x_8 x_9} = 1$	$-R$	11	$\eta_{x_1 x_2^2 x_3^2 x_5 x_6 x_7 x_8 x_{10} x_{11} x_{12} x_{13}} = 1$	$-(k_{7r} + k_2 + k_{2'})(k_{8r} + k_4)$
8	$\eta_{x_1 x_2 x_3 x_4^2 x_5 x_6 x_7 x_8} = 1$	$\frac{\sigma}{2}$	11	$\eta_{x_1^2 x_2 x_3^2 x_5 x_6 x_7 x_8 x_{10} x_{11} x_{12} x_{13}} = 1$	$(k_{7r} + k_4)(k_{8r} + k_4)$
8	$\eta_{x_1 x_2 x_3^2 x_4 x_5 x_6 x_7 x_9} = 1$	$b_2 \sigma$	11	$\eta_{x_1^2 x_2^2 x_3 x_5 x_6 x_7 x_8 x_{10} x_{11} x_{12} x_{13}} = 1$	$-(k_{7r} + k_4)(k_{8r} + k_{6'})$
8	$\eta_{x_1^2 x_2 x_3 x_4 x_5 x_6 x_8 x_9} = 1$	$b_2 \sigma$	11	$\eta_{x_2 x_3^2 x_4 x_5 x_6 x_7 x_8^2 x_{10} x_{11} x_{12} x_{13}} = 1$	$k_{\mathrm{wee}}(k_{8r} - k_4)$
8	$\eta_{x_1 x_2^2 x_3 x_4 x_5 x_6 x_7 x_8} = 1$	$-\frac{\sigma}{2}$	11	$\eta_{x_2^2 x_3 x_4 x_5 x_6 x_7 x_8^2 x_{10} x_{11} x_{12} x_{13}} = 1$	$k_{\mathrm{wee}}(k_{8r} + k_{6'})$
5	$\eta_{x_1^2 x_2 x_3^2 x_4^3 x_5} = 0.90$	$-\frac{b_2^2 \sigma^4}{2}(x_2 - x_4)$	11	$\eta_{x_2^2 x_3 x_5 x_6 x_7 x_8^2 x_9 x_{10} x_{11} x_{12} x_{13}} = 1$	$k_{\mathrm{wee}}(k_{7r} + k_2 + k_{2'})$
			9	$\eta_{x_1^2 x_2^2 x_3^2 x_5 x_6 x_7 x_{11} x_{12}^2 x_{13}} = 0.93$	$\frac{(k_{7r}+k_2+k_{2'})(k_{8r}+k_4)(x_{12}-1)(k_{7r}+k_4)}{K_{\mathrm{mc}}+1-x_{12}} k_{\mathrm{c}} \beta$
